# SYK-mediated epithelial cell state is associated with response to c-Met inhibitors in c-Met-overexpressing lung cancer

**DOI:** 10.1038/s41392-023-01403-w

**Published:** 2023-05-15

**Authors:** Ji Zhou, Xu-Chao Zhang, Shan Xue, Mengdi Dai, Yueliang Wang, Xia Peng, Jianjiao Chen, Xinyi Wang, Yanyan Shen, Hui Qin, Bi Chen, Yu Zheng, Xiwen Gao, Zuoquan Xie, Jian Ding, Handong Jiang, Yi-Long Wu, Meiyu Geng, Jing Ai

**Affiliations:** 1 Division of Antitumor Pharmacology, State Key Laboratory of Drug Research, Shanghai Institute of Materia Medica, Chinese Academy of Sciences, Shanghai, 201203 China; 2 University of Chinese Academy of Sciences, No. 19A Yuquan Road, Beijing, 100049 China; 3 Guangdong Provincial Key Laboratory of Translational Medicine in Lung Cancer, Guangdong Lung Cancer Institute, Guangdong Provincial People’s Hospital, and Guangdong Academy of Medical Sciences, School of Medicine, South China University of Technology, Guangzhou, 510080 China; 4 Department of Respiratory Medicine, Ren Ji Hospital, School of Medicine, Shanghai Jiao Tong University, Shanghai, 200127 China; 5 Department of Neurobiology, Brain Institute, University of Pittsburgh, Pittsburgh, 15213 USA; 6 Department of Respiratory Medicine, Affiliated Hospital of Xuzhou Medical University, Xuzhou, 221000 China; 7 Department of Respiratory Medicine, Minhang Hospital, Fudan University, Shanghai, 201199 China; 8 School of Pharmaceutical Science and Technology, Hangzhou Institute for Advanced Study, University of Chinese Academy of Sciences, Hangzhou, 310024 P. R. China

**Keywords:** Lung cancer, Predictive markers

## Abstract

Genomic *MET* amplification and exon 14 skipping are currently clinically recognized biomarkers for stratifying subsets of non-small cell lung cancer (NSCLC) patients according to the predicted response to c-Met inhibitors (c-Metis), yet the overall clinical benefit of this strategy is quite limited. Notably, c-Met protein overexpression, which occurs in approximately 20–25% of NSCLC patients, has not yet been clearly defined as a clinically useful biomarker. An optimized strategy for accurately classifying patients with c-Met overexpression for decision-making regarding c-Meti treatment is lacking. Herein, we found that SYK regulates the plasticity of cells in an epithelial state and is associated with their sensitivity to c-Metis both in vitro and in vivo in PDX models with c-Met overexpression regardless of *MET* gene status. Furthermore, TGF-β1 treatment resulted in SYK transcriptional downregulation, increased Sp1-mediated transcription of FRA1, and restored the mesenchymal state, which conferred resistance to c-Metis. Clinically, a subpopulation of NSCLC patients with c-Met overexpression coupled with SYK overexpression exhibited a high response rate of 73.3% and longer progression-free survival with c-Meti treatment than other patients. SYK negativity coupled with TGF-β1 positivity conferred *de novo* and acquired resistance. In summary, SYK regulates cell plasticity toward a therapy-sensitive epithelial cell state. Furthermore, our findings showed that SYK overexpression can aid in precisely stratifying NSCLC patients with c-Met overexpression regardless of *MET* alterations and expand the population predicted to benefit from c-Met-targeted therapy.

## Introduction

Targeted therapies have greatly improved clinical outcomes and reshaped the treatment standards for patients with genomic alterations in *EGFR, ALK, ROS1, NTRK*, etc. The response rate to EGFR inhibitors (EGFRis) is approximately 70% in patients with *EGFR* mutations,^1,2^ who account for 15–40% of patients with non-small cell lung cancer (NSCLC). However, the scenario in patients with *MET* alterations is quite different. c-Met alterations in lung cancer include gene mutation, amplification and protein overexpression.^3,4^ Recent studies reported a response rate of 40–70% in patients with *MET* exon 14 skipping mutation (*MET ex14Δ*).^5–7^ However, such *MET* alterations are only present in a rare subtype of NSCLC, accounting for only 3–4% of NSCLC cases.^5,6^ A much more limited response was observed in patients with *MET* amplification^8–10^ or c-Met overexpression.^11,12^ While c-Met overexpression occurs in approximately 20–25% of NSCLC patients,^3,13,14^ it has not yet been clearly defined as a clinically useful biomarker. Efforts to identify predictive biomarkers, especially in the c-Met protein overexpression subtype, might help precisely expand the population that would benefit from the clinical use of c-Met inhibitors (c-Metis).

With advancement in research on cancer mechanisms, phenotypic plasticity and disrupted differentiation have been recognized as discrete hallmarks that are highly involved in sensitivity to anticancer therapy.^15–17^ Epithelial-mesenchymal transition (EMT) is recognized as one of the best examples of cancer cell plasticity; EMT is the process by which epithelial cancer cells dedifferentiate into a highly motile and mesenchymal tumor phenotype. It is also an important mechanism for inducing resistance to kinase-targeted therapy.^16,18^ Generally, an epithelial state rather than a mesenchymal state is more often associated with therapeutic response to kinase-targeted therapy.^16,17,19^ Therefore, key factors that inhibit EMT have the potential to become new sensitive predictive biomarkers that, when combined with existing therapies, may lead to better and more durable clinical responses. Here, we hypothesized that such factors facilitating the epithelial cell state might also account for the sensitivity to c-Metis in the c-Met-overexpressing patient subset.

Spleen tyrosine kinase (SYK) is a cytoplasmic kinase that couples immune cell receptors to induce intracellular signaling pathways and plays a central role in adaptive and inflammatory immune responses.^20^ SYK plays a crucial role in hematological malignancies, particularly B-cell lineage lymphomas. Accumulating data also suggest that SYK is involved in carcinogenesis and tumor progression in solid tumors, acting as either a tumor-promoting or tumor-suppressive factor according to the biological context and tissue lineage.^20,21^ Here, we assessed the epithelial cell state and performed comprehensive omics analysis in c-Met-overexpressing cell lines, with lung cancer cell lines harboring EGFR mutations as positive controls, as the appearance of mesenchymal features is well recognized to be associated with resistance to EGFR inhibitors^16,17,22–26^; we validated the results in patient-derived xenograft (PDX) models and NSCLC patient cohorts. We found that SYK regulated the epithelial cell state, enabling the selection of SYK-high NSCLC patients in the c-Met-overexpressing group as a population that may benefit from c-Meti therapy.

## Results

### SYK maintains the epithelial cell state and is associated with sensitivity to both c-Metis and EGFRis

**Fig. 1:**
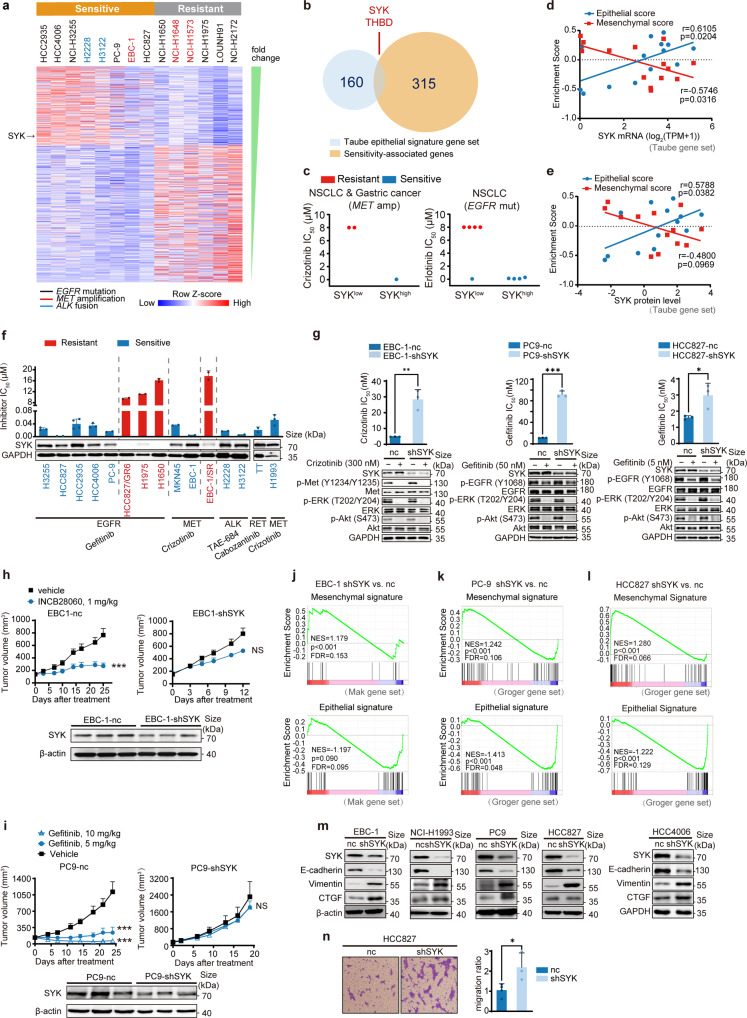
High SYK expression, which maintains the epithelial state, is associated with the sensitivity to kinase inhibitors. **a** Heatmap showing normalized mRNA expression of significantly differentially expressed genes (*p* < 0.02, log2 (fold change) >2 and FDR < 0.25) in 14 kinase-addicted cancer cell lines classified as sensitive or resistant (as determined by IC_50_) to specific kinase inhibitors. **b** Venn diagram illustrating the overlap of epithelial signature genes (Taube gene set) with the significant sensitivity-associated genes indicated in Fig. 1a. **c** The association between SYK expression and the response to the indicated kinase inhibitor. The median value of mRNA expression was used as the cutoff for the classification of high and low SYK expression. **d**, **e** Pearson correlation analysis b**e**tween epithelial/mesenchymal (E/M) enrichment scores and SYK mRNA (**d**) and protein levels (**e**) in the cell lines indicated in Fig. 1a. Panel **e** does not display the NCI-H3122 c**e**ll line data since SYK protein level data for NCI-H3122 cells are not available in the CCLE. **f** Cell sensitivity to the indicated kinase inhibitors was stratified according to SYK expression in various cancer cells harboring different genetic aberrations. These cell lines included 13 NSCLC cell lines, 1 *MET*-amplified gastric cancer cell line (MKN45) and 1 *RET*-mutant thyroid carcinoma cell line (TT) to represent the typical molecular alterations of NSCLC. Detailed information on these cancer cell lines is listed in Supplementary Table S2. Semiquantification of SYK relative to GAPDH and further normalization relative to EBC-1 is shown. **g** The change in cell sensitivity upon stable SYK knockdown. **h** The change in the response to INCB28060 in EBC-1 xenograft-bearing mice upon stable SYK knockdown (n = 6/group in EBC-1-shSYK model, *n* = 7/group in EBC-1-nc model). **i** The change in the response to gefitinib in PC9 xenograft-bearing mice upon stable SYK knockdown (*n* = 6 for each group). **j** to **l** GSEA of stable SYK knockdown versus negative control EBC-1 (**j**), PC-9 (**k**) and HCC827 (**l**) cells. The gene sets shown are indicated as epithelial or mesenchymal state-associated genes. FDR, false discovery rate. NES, normalized enrichment score. **m** Immunoblotting analysis of representative E/M signatures in cell lines upon stable SYK knockdown. **n** The change in cell migration upo**n** stable SYK knockdown. The data shown are representative results from three independent experiments in **g**, **m**, **n**, and from two to four independent experiments in **f**. The data in **f**, **g** and **n** are presented as the mean ± SD; the data in **h**, **i** are presented as the mean ± SEM. ***p < 0.001, **p < 0.01, and *p < 0.05 using Student’s *t* test in **g**, **h** and **n**, or one-way ANOVA in **i**

We first used the public Cancer Cell Line Encyclopedia (CCLE) database^27^ and profiled 14 lung cancer cell lines harboring *MET* amplification with *EGFR* mutation or *ALK* translocation as positive controls. We found that 315 genes were significantly associated with sensitivity to c-Metis, EGFRis and ALK inhibitors (ALKis) at the transcriptomic level (Fig. 1a). We then selected four featured signatures^24,28–30^ as representative of a highly epithelial state. We identified the intersecting genes among the above 315 genes as *SYK*, *THBD*, *RAPGEF5*, and *MUC1* (Fig. 1b and Supplementary Fig. S1a-c). SYK, a cytoplasmic kinase well known for its ability to couple immune cell receptors,^20^ was chosen for further analysis. We found that SYK expression at both the mRNA and protein level was significantly higher in the sensitive group than in the resistant group (Supplementary Fig. S1d). We then focused only on *MET*-amplified samples with *EGFR*-mutant samples as a control. We found that cancer cell lines with high SYK expression showed sensitivity to inhibition of both c-Met and EGFR. However, cancer cell lines with low SYK expression failed to show a drug response (Fig. 1c). Together, these data indicate that SYK plays a critical role in determining sensitivity to c-Meti and EGFRi treatment.

To address whether SYK-associated sensitivity was mediated by the epithelial cell state, we first assigned samples an epithelial score and a mesenchymal score using gene set variation analysis (GSVA)^31^ with four individual epithelial/mesenchymal signature gene sets^24,28–30^ (Taube, Groger, Mak, and Byers) (Supplementary Table S1). We then performed Pearson correlation analysis between epithelial/mesenchymal scores and SYK protein/mRNA levels. Notably, SYK mRNA and protein levels showed a consistent and relatively strong positive correlation with epithelial scores (Pearson r: 0.5788–0.7374), and the correlation was significant (*p* < 0.05) (Fig. 1d, e, and Supplementary Fig. S1e, f). However, SYK expression was not significantly correlated with some types of mesenchymal scores in some analyses (SYK protein level vs. Taube mesenchymal score, SYK level vs. Byers mesenchymal score) and/or showed a lower negative correlation with mesenchymal score in some analyses (SYK level vs. Byers mesenchymal score) (Fig. 1d, e, and Supplementary Fig. S1e, f). All these results suggest that SYK is associated with sensitivity to c-Metis and EGFRis, which is more closely associated with maintaining the epithelial cell state. Other typical epithelial/mesenchymal signature markers, including *CDH1*, *VIM*, *CDH2*, and *TGFB1*, failed to individually predict a drug response to c-Metis and EGFRis (Supplementary Fig. S2).

We then assessed the association of SYK with the therapeutic response to c-Metis, EGFRis, and other kinase inhibitors for the treatment of NSCLC, such as ALKis and RET inhibitors (RETis), in 15 cancer cell lines (Fig. 1f and Supplementary Table S2). Cancer cells with basal levels of SYK expression characterized by visible bands (values ≥ 0.5) were sensitive to all four types of kinase inhibitors, whereas cancer cells with faint bands (values ≤ 0.1) consistently exhibited resistance to these inhibitors (Fig. 1f and Supplementary Fig. S3a). These results strongly suggest that high SYK expression can predict the therapeutic response to kinase inhibitors in kinase-addicted cell settings.

For confirmation, we used an shRNA-based approach to deplete the expression of SYK in the *MET*-amplified EBC-1 cell line, *EGFR*-mutant PC-9 cells, HCC827 cells and *ALK*-translocated H2228 cells. We found that SYK knockdown led to decreased sensitivity to c-Metis, EGFRis, and ALKis (Fig. 1g and Supplementary Fig. S3b, c). Consistently, in the in vivo xenograft model, compared to control mice, mice bearing *MET*-amplified EBC-1 lung cancer cells with stable SYK knockdown exhibited a significantly reduced response to the c-Met inhibitor INCB28060 (Fig. 1h). Similar results were observed in another two tested models, one model involving *MET*-amplified MKN45 cancer cells with stable SYK knockdown and one model involving *EGFR*-mutant PC9 cells with stable SYK knockdown (Fig. 1i and Supplementary Fig. S3d). We then explored whether SYK depletion-induced resistance to c-Meti and EGFRi treatment was mediated by a mesenchymal state. We performed RNA sequencing (RNA-seq) analysis in the *MET*-amplified EBC-1 cell line and *EGFR*-mutant PC-9 and HCC827 cell lines upon SYK knockdown. We performed gene set enrichment analysis (GSEA) of RNA-seq data comparing the SYK shRNA group to the nc control group, and we found that SYK depletion was positively associated with the mesenchymal signature (NES = 1.179 to 1.365, FDR < 0.25) but negatively associated with the epithelial signature (NES = −1.413 to −1.153, FDR < 0.25) (Fig. 1j–l and Supplementary Fig. S4a-c). Real-time quantitative reverse transcription PCR (qRT‒PCR) further confirmed that SYK knockdown led to decreased expression of epithelial cell markers and increased expression of mesenchymal cell markers (Supplementary Fig. S4d-f). Further immunoblotting of representative epithelial or mesenchymal markers showed that stable SYK knockdown influenced the expression of two typical canonical EMT markers: it increased vimentin levels and decreased E-cadherin levels in all five tested cancer cell lines with *MET* amplification or *EGFR* mutation. In addition, CTGF was upregulated in *EGFR*-mutant H4006 cells, PC-9 cells, *MET*-amplified EBC-1 cells, and NCI-H1993 cells upon SYK knockdown, while it was minimally influenced by SYK knockdown in *EGFR*-mutant HCC827 cells (Fig. 1m), indicating that the use of CTGF for the characterization of EMT has certain limitations. It is therefore not surprising that while CTGF induces EMT-like changes,^32^ it is generally not listed as a general canonical marker in EMT studies.^33,34^ Furthermore, the SYK-depleted cells became highly migratory, substantiating the key role of SYK in EMT (Fig. 1n).

### SYK interacts with Sp1 to suppress Sp1-activated FRA1 transcription, conferring sensitivity to c-Metis and EGFRis

**Fig. 2:**
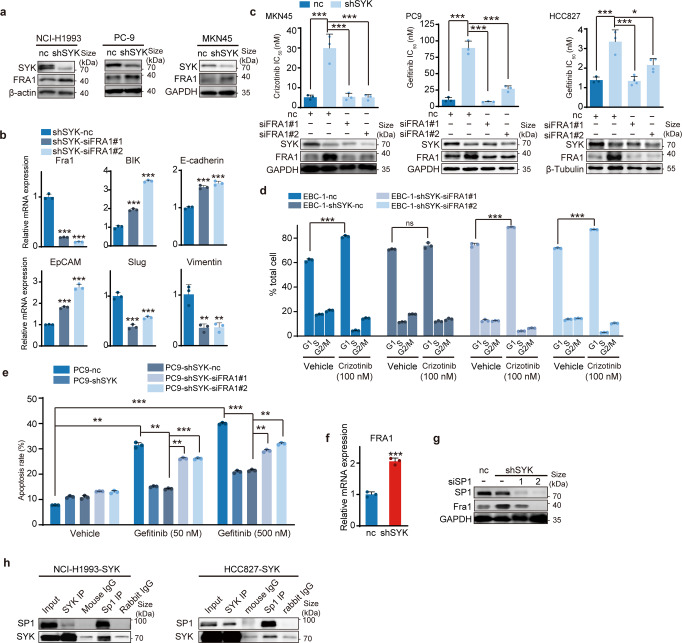
SYK interacts with Sp1, suppressing Sp1-induced FRA1 transcription and conferring sensitivity to kinase inhibitors. **a** Immunoblotting analysis of FRA1 in cell lines upon stable SYK knockdown. **b** qRT‒PCR analysis of the representative E/M classic markers in HCC827-shSYK cells upon transient transfection with FRA1 siRNA. **c** Change in drug sensitivity in the indicated *MET*-amplified or *EGFR*-mutant cells with stable SYK knockdown upon further siFRA1 transient transfection. **d** Change in the cell cycle arrest of EBC-1-shSYK cells upon siFRA1 transient transfection. **e** Change in the apoptosis rate of PC9-shSYK cells upon siFRA1 transient transfection. **f** qRT‒PCR analysis of FRA1 transcriptional levels upon stable SYK knockdown in the HCC827 cell line. **g** Immunoblot analysis of FRA1 in PC9-shSYK cells upon Sp1 transient transfection. **h** Whole-cell lysates (WCLs) of SYK ectopically overexpressing NCI-H1993 or HCC827 cells were immunoprecipitated with antibodies against SYK or Sp1. Rabbit IgG antibody and mouse IgG antibody were used as negative controls for immunoprecipitation, and WCLs and coimmunoprecipitation were detected by immunoblotting, as indicated. The data shown are representative results from two or three independent experiments. The data in **b**, **c**, **d**, **e**, **f** are presented as the mean ± SD ***p < 0.001, **p < 0.01 and *p < 0.05, using one-way ANOVA in **b**, **c** and **e**; using Student’s *t* test in **d** and **f**

Furthermore, we identified the key downstream regulator of SYK that determines the epithelial cell state associated with sensitivity to c-Metis and EGFRis. A previous report^35^ indicated that in breast cancer cells, SYK was able to downregulate the transcription factor FRA1, which acts as a key EMT switch as well as a drug resistance mediator in cancer.^36^ We then investigated whether FRA1 was downstream of SYK in our scenario. We found that depletion of SYK increased FRA1 expression in representative cell lines with *MET* amplification or *EGFR* mutation (Fig. 2a), indicating that SYK could negatively regulate FRA1. Furthermore, we used siRNA to reduce FRA1 expression in SYK stable knockdown cells and showed increased mRNA expression of canonical epithelial cell markers and decreased expression of mesenchymal cell markers (Fig. 2b). Similarly, further FRA1 knockdown reversed SYK depletion-induced downregulation of E-cadherin protein and upregulation of Vimentin protein (Supplementary Fig. S5a). These results indicated that FRA1 depletion largely reversed the SYK depletion-induced mesenchymal state. Accordingly, FRA1 knockdown reversed the reduction in both c-Meti and EGFRi sensitivity induced by SYK depletion (Fig. 2c). In addition, it’s known that response to c-Meti and EGFRi is associated with a G0/G1 cell cycle arrest^37^ and cell apoptosis,^38^ respectively. Correspondingly, SYK depletion in MET-amplified cancer cells reduced the G0/G1 cell cycle arrest-induced by c-Meti, while further FRA1 knockdown reversed this reduction (Fig. 2d and Supplementary Fig. S5b). Similar trend was observed in EGFRi-induced cell apoptosis effect (Fig. 2e). These findings suggest that SYK determines sensitivity to both c-Metis and EGFRis by downregulating FRA1.

Next, we explored the regulatory mechanism of SYK on FRA1. SYK interacts with the transcription factor Sp1 and then suppresses Sp1-activated FRA1 gene transcription.^35^ We found increased FRA1 transcription in SYK stable knockdown cells (Fig. 2f). Sp1 knockdown with siRNA strongly reversed SYK-knockdown-induced FRA1 upregulation at both the protein and mRNA levels (Fig. 2g and Supplementary Fig. S5c). Then, we carried out coimmunoprecipitation experiments to determine whether SYK and Sp1 are physically associated. Endogenous Sp1 was immunoprecipitated with an anti-SYK antibody from the cell lysates of NCI-H1993 cells transiently expressing SYK, and vice versa (Fig. 2h). Similar results were also obtained in EGFR-mutant HCC827 cells stably expressing SYK (Fig. 2h). These data demonstrated a significant interaction between SYK and Sp1. All these results indicated that mechanistically, SYK interacts with Sp1 and then suppresses Sp1-induced transcription of FRA1, the key EMT switch and drug resistance mediator.

### TGF-β1 downregulates SYK via Smad2/3 and confers resistance to c-Metis and EGFRis

**Fig. 3:**
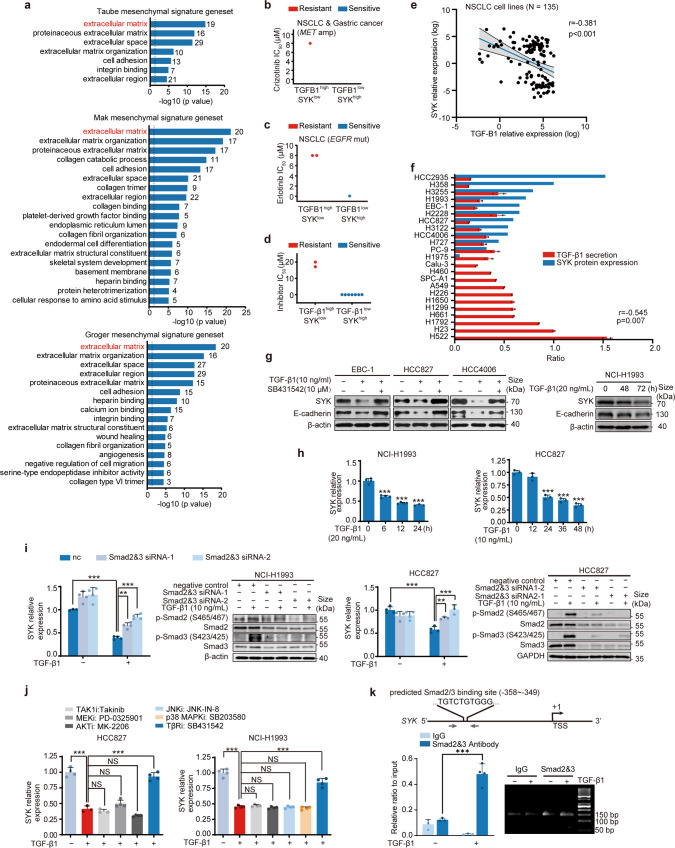
Transcriptional downregulation of SYK by TGF-β1 confers resistance to c-Metis and EGFRis. **a** GO annotation and enrichment analysis of mesenchymal signature gene sets (p < 0.05, FDR < 0.05). Genes in the extracellular matrix gene set are shown. **b** and **c** Samples were stratified based on crizotinib (**b**) or erlotinib (**c**) sensitivity according to the SYK/TGF-β1 mRNA expression pattern based on the data from CCLE. **d** A panel of available cancer cell lines (also seen in Fig. 1f) was stratified based on cell sensitivity to kinase inhibitors according to the SYK/TGF-β1 expression pattern. The secreted TGF-β1 level was normalized to the total cellular protein level. Samples were classified into high and low TGF-β1 subgroups using the median value as the cutoff in **b**-**d**. **e** Pearson correlation analysis between TGF-β1 and SYK mRNA expression in NSCLC cell lines based on the data from CCLE. **f** The association between TGF-β1 secretion and SYK protein levels in the profiled NSCLC cell lines. **g** Immunoblot analysis of the indicated cell lines treated with TGF-β1 alone or together with TGF-β1 inhibitor for 96 h or the indicated time. E-cadherin was investigated as a functional indicator of TGF-β1. **h** SYK mRNA level alterations in NCI-H1993 (left) or HCC827 (right) cells treated with TGF-β1. **i**, **j** Alteration of SYK transcription levels upon Smad2/3 knockdown (**i**) or treatment with the indicated inhibitors (**j**). **k** Smad2/3 enrichment in the SYK promoter measured by a ChIP assay. Upper: schematic diagram of SYK promoter showing location of putative Smad2/3 binding sites. The positions of the ChIP primers are delineated by gray arrowheads. TSS, transcription start site. Lower: qRT-PCR analysis (left) and agarose gel electrophoresis detection (right) by the ChIP assay. The data shown are representative results from two or three independent experiments in **f–k**. NS: no significance, ***p < 0.001, **p < 0.01, using Student’s *t* test in **k**, using one-way ANOVA in **h**, **i**, **j**

As shown in the right panel of Fig. 1c, the response to EGFRi in the SYK^low^ subtype was inconsistent (some resistant cells and some sensitive cells), which indicated the limitation of low SYK expression alone in predicting resistance. Since a highly mesenchymal cell state is commonly associated with resistance,^15^ we assessed a mesenchymal signature gene set by Gene Ontology (GO) enrichment analysis. We found that the extracellular matrix term gene set, including *LTBP1/2*, *TGF-Β1I1*, *BGN*, *DCN* and *SERPINE1/2*, was the top-ranked gene set (Fig. 3a and Supplementary Table S3). Interestingly, these genes are all known to be related to TGF-β1 signaling.^39,40^ Given that TGF-β1 is a key inducer of the mesenchymal state and is known to be involved in resistance to targeted therapy,^19^ we examined whether TGF-β1 expression could predict drug resistance. We found that TGF-β1 expression alone failed to predict drug resistance (Supplementary Fig. S2f and Supplementary Fig. S6). However, high TGF-β1 expression coupled with low SYK expression could be used to predict *de novo* resistance to either c-Metis or EGFRis in the CCLE database or in the representative cancer cell lines (Fig. 3b–d). Furthermore, we found a negative correlation between SYK and TGF-β1 at both the mRNA and protein levels (Fig. 3e, f). Mechanistic studies demonstrated that TGF-β1 exposure led to significant downregulation of SYK and upregulation of FRA1 along with a shift to a mesenchymal cell state in *MET*-amplified and *EGFR*-mutant NSCLC cell lines, and these effects were reversed by a TGF-β1 inhibitor (Fig. 3g and Supplementary Fig. S7a). These results collectively suggest that TGF-β1-mediated downregulation of SYK led to an enhanced mesenchymal state, enabling the prediction of drug resistance. Likewise, reduced SYK expression with increased TGF-β1 secretion as well as increased FRA1 expression was noted in cells with acquired resistance to c-Metis and EGFRis (Supplementary Fig. S7b).

We then investigated the mechanism by which TGF-β1 downregulates SYK. A significant reduction in SYK mRNA expression was observed upon exposure to TGF-β1 (Fig. 3h and Supplementary Fig. S7c), and this SYK reduction was not reversed by either a proteasome inhibitor or a protease inhibitor (Supplementary Fig. S7d, e), indicating that TGF-β1 regulates the transcription of SYK. We used both siRNA and inhibitors to block the major canonical and noncanonical effectors downstream of TGF-β1. We found that only Smad2/3 knockdown reversed the reduction in SYK levels in response to TGF-β1 (Fig. 3i, j). We next applied a chromatin immunoprecipitation (ChIP) assay, and we observed that TGF-β1 treatment led to the recruitment of Smad2/3 to the SYK promoter (Fig. 3k). Collectively, these results suggest that TGF-β1 activates Smad2/3, which binds to the promoter region of SYK and consequently leads to transcriptional suppression of SYK.

### High SYK expression indicates a therapeutic response in PDX models

**Fig. 4:**
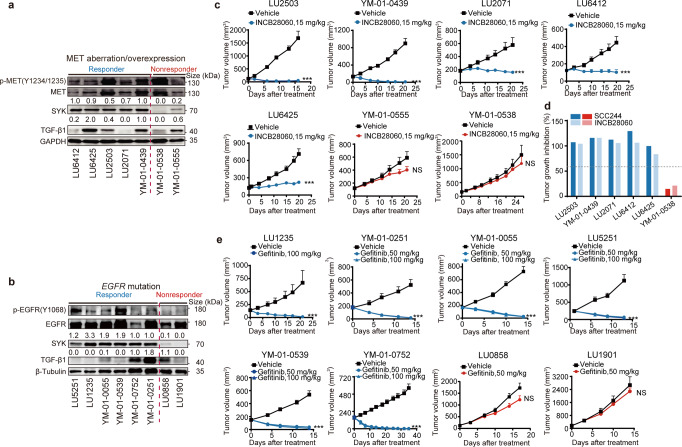
SYK-high status indicates a therapeutic response to c-Metis and EGFRis in NSCLC PDX models. **a** and **b** Intratumoral expression of SYK and TGF-β1 in 15 primary lung cancer PDX models harboring c-Met (**a**) and EGFR (**b**) aberrations prior to treatment. Detailed information on these PDX models is listed in Supplementary Table S4. The expression level was semiquantified and normalized to that of the GAPDH control. The values above the immunoblots indicate the SYK and TGF-β1 levels in each model relative to those in the LU-01-0439 (c-Met panel) and LU-01-0752 (*EGFR* panel) models. SYK values ≥ 1.0 and 0.5 were defined as high, while SYK values ≤ 0.1 and 0.2 were defined as low in the *EGFR* panel and c-Met panel, respectively. The median TGF-β1 expression was used as the cutoff for the classification of samples into high and low expression groups. **c** and **e** Tumor volume curves for PDX models treated with the indicated kinase inhibitor at the indicated doses. **d** Tumor growth inhibition in PDX models treated with c-Meti SCC244 or capmatinib. Error bars represent the mean ± SEM (at least 6 mice per group)

Next, we started a small-scale PDX-based parallel trial to assess the ability to stratify subjects with SYK high expression or low SYK expression plus high TGF-β1 expression into response or nonresponse groups in vivo. We screened commercially available NSCLC PDXs from HuBase and preferentially focused on models harboring c-Met aberrations or *EGFR* mutations. Fifteen PDX models, including 8 EGFR-mutant and 7 c-Met-dysregulated models, were included (Supplementary Table S4). Intratumoral expression of both SYK and TGF-β1 was examined using immunoblotting and was semiquantified by densitometric analysis prior to the treatment (Fig. 4a, b, values above the indicated immunoblots). Mice were treated with c-Meti (capmatinib/INCB28060 or SCC244^41^) or EGFRi (gefitinib) once a day consecutively (Fig. 4c–e). We used 60% as the endpoint tumor growth inhibition (TGI) rate cutoff. Highly consistent with the in vitro data, 5 out of the 7 c-Met-aberrant PDX models had high SYK expression (SYK value ≥ 0.5) and showed a favorable response to c-Metis (Fig. 4a, c and d, Supplementary Fig. S8a, and Supplementary Table S4). Notably, 2 of the 4 c-Met-overexpressing PDX models without *MET* amplification had high SYK expression and responded well to c-Metis, while the other 2 models with low SYK expression, in which the one with high TGF-β1 expression (SYK value ≤ 0.2, TGF-β1 value ≥ 0.6) exhibited resistance (YM-01-0555) (Fig. 4a, c). These findings suggest that patients with c-Met overexpression can be substratified according to the SYK expression level; thus, SYK may represent a new, sensitive biomarker for predicting the response to c-Meti treatment. Similar results were also observed in *EGFR*-mutant PDX models (Fig. 4b, e, and Supplementary Fig. S8b). To verify whether reduced SYK expression coupled with increased TGF-β1 expression could predict the acquisition of drug resistance, EGFR-mutant PDX models with induced acquired resistance to erlotinib (YM-01-0055R) and the parental erlotinib-sensitive YM-01-0055 PDX model were used. Encouragingly, decreased SYK expression coupled with increased TGF-β1 expression was uniformly observed in the acquired resistance models (Supplementary Fig. S8c, d).

### Patients with SYK-positive/EGFR-mutant NSCLC uniformly respond to EGFRis

To explore the translational implications, we first used an EGFRi-treated NSCLC cohort as a proof-of-principle cohort and to define the criteria for SYK-positive status. This cohort included 31 patients with advanced *EGFR* mutation-positive NSCLC who received first-generation EGFRis (Supplementary Table S5). For this analysis, we defined patients with a complete response (CR) or PR as responders and those with progressive disease (PD) as nonresponders (*de novo* resistance). The receiver operating characteristic (ROC) curve showed that the optimum diagnostic cutoff score for SYK was 2, and this score had a specificity of 100% (Supplementary Fig. S9a). Using this cutoff criterion, we found that when patients were stratified by SYK status and EGFR status, the 21 patients with high SYK expression and EGFR mutation showed a uniform therapeutic response to EGFRis, with a response rate of 100% (Supplementary Fig. S9b-e), which is much higher than the 74.2% response rate of patients stratified by *EGFR* mutation alone (23 PR and 8 stable disease (SD)).

### Concurrent expression of SYK and c-Met reveals clinical benefits in the c-Met-overexpressing NSCLC subset

**Fig. 5:**
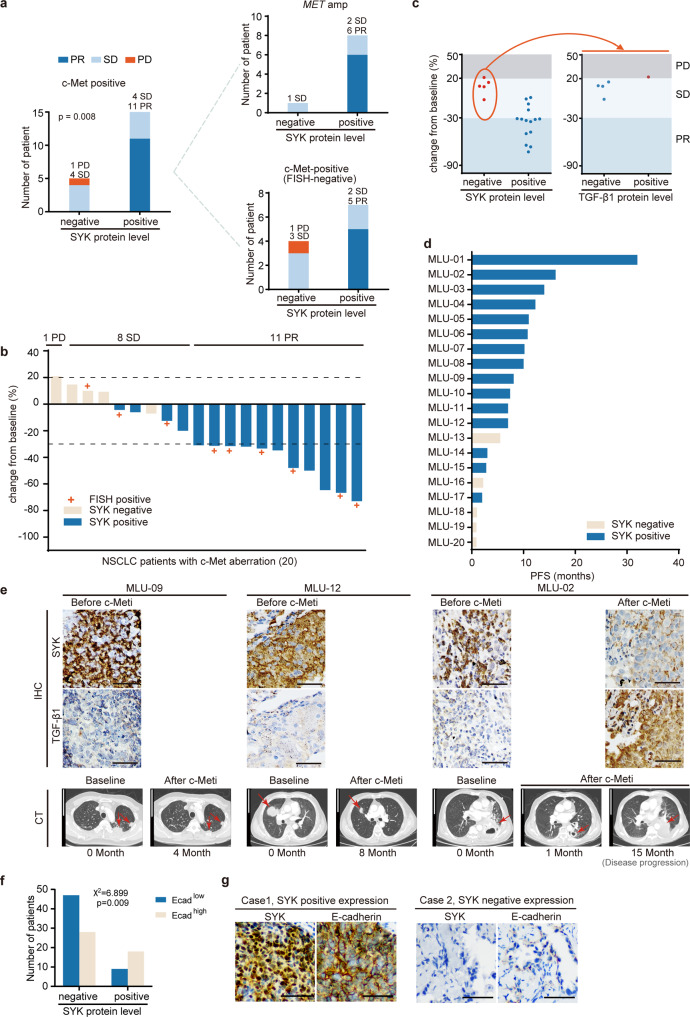
Clinical applications of the SYK-positive subtype and the SYK-negative/TGF-β1-positive subtype in c-Met-positive NSCLC patients receiving c-Met-targeted therapy. **a** Distribution of the therapeutic response of c-Met-positive patients classified as SYK-positive and SYK-negative. **b** Waterfall plot of the maximum change from baseline of the longest tumor diameter for patients with evaluable tumors in the c-Met-positive cohort. **c** Patients treated with the indicated c-Meti (as determined by the maximum decrease) were stratified according to SYK expression (positive vs. negative) (left) based on IHC analysis to predict the treatment response. In the subgroup with negative SYK expression, patients treated with the indicated c-Meti were further stratified by TGF-β1 expression (right) to predict the treatment response. **d** PFS results (months) obtained with the indicated c-Meti in c-Met-aberrant subgroups. **e** Representative SYK and TGF-β1 staining (upper) and chest CT images of patients before and after targeted therapy (lower). Detailed case information is presented in the Methods section. The arrow indicates the location of the tumor. Scale bars for the IHC image, 50 μm. Scale bars for the CT image, 10 cm. Clinical assessment was performed based on RECIST, version 1.1. Patients with a CR or PR were considered responders, while patients with PD were considered nonresponders (*de novo* resistance). **f** Crosstab analysis with Pearson chi-square test of E-cadherin and SYK expression in 102 NSCLC specimens. **g** Representative IHC images of SYK and E-cadherin staining. Scale bars, 50 μm

We next assessed the c-Met dysregulated cohort with the same criteria defined in the EGFR subgroup analysis. An independent cohort of 20 NSCLC patients with c-Met positivity with or without *MET* amplification (11 and 9, respectively) who were treated with the c-Meti crizotinib or capmatinib was selected (Supplementary Table S6). Tumor tissues obtained before treatment were analyzed by IHC. We found that all patients who were both c-Met-positive and SYK-positive attained significant clinical benefit (11 PR and 4 SD), with a response rate of 73.3% (Fig. 5a-c). Notably, in 11 patients with c-Met overexpression but without *MET* amplification, five SYK-positive patients exhibited significant tumor shrinkage after treatment with c-Metis (Fig. 5a, b). Moreover, patients who were SYK-positive tended to have longer progression-free survival (PFS) than patients who were SYK-negative (Fig. 5d). SYK and TGF-β1 staining and chest CT images of representative patients before and after targeted therapy are shown in Fig. 5e.

*De novo* resistance to c-Metis was found in SYK-negative/TGF-β1-positive patients (Fig. 5c). Moreover, of the 3 patients with acquired resistance to c-Metis after an initial response, SYK expression was decreased and TGF-β1 expression was increased in 1 of these patients (Fig. 5e, MLU-02).

E-cadherin is a canonical epithelial hallmark, and loss of E-cadherin expression is a hallmark of EMT.^42,43^ We then tested both the E-cadherin level and SYK level in 102 NSCLC specimens by IHC to explore whether high SYK expression was associated with the epithelial phenotype in patient samples. We found that positive SYK expression was significantly associated with a high level of E-cadherin expression (*p* = 0.009, Pearson’s chi-squared test); 66.7% of patients with positive SYK expression exhibited high E-cadherin expression (Fig. 5f). The corresponding representative IHC images showing E-cadherin and SYK expression are in Fig. 5g. These data suggest that high SYK expression is significantly associated with the epithelial phenotype in patient samples, represented by the expression of the classical epithelial marker E-cadherin.

## Discussion

**Fig. 6:**
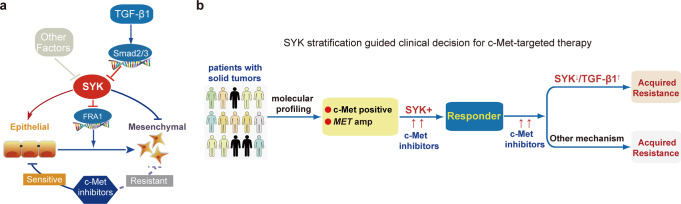
Proposed working scheme of SYK. **a** The proposed mechanism by which SYK affects the therapeutic response to c-Metis. SYK regulates the epithelial cell state and is associated with the therapeutic response to c-Metis, while SYK downregulation by TGF-β1 is associated with an enhanced mesenchymal state that confers resistance to c-Metis. **b** A proposed scheme of SYK stratification to guide clinical decisions related to c-Met-targeted therapy

In the present study, for the first time, we revealed that the epithelial plasticity induced by SYK dictates the response to c-Meti in a c-Met-overexpressing subset of patients and opens a new avenue for expanding the population predicted to benefit from c-Meti treatment among patients with c-Met overexpression. Mechanistic insights revealed that SYK interacts with Sp1 and then suppresses Sp1-induced transcription of FRA1, the key EMT switch and drug resistance mediator.^36^ Furthermore, we found that TGF-β1-activated Smad2/3 directly binds to the promoter region of SYK and in turn suppresses the transcription of SYK; this effect was associated with an enhanced mesenchymal state, which facilitated resistance to c-Metis (Fig. 6a).

*MET ex14Δ* and amplification confer sensitivity to c-Metis and are clinically used as sensitivity biomarkers. c-Met selective inhibitors have been approved for the treatment of NSCLC patients with *MET ex14Δ* in recent years. Other factors that influence the responsiveness to c-Metis in *MET*-dysregulated cancer have been gradually found. Similar to the mechanisms of resistance to other kinase inhibitors, the reported mechanisms of primary or acquired resistance to c-Metis include on-target *MET* mutations, amplification of the *MET* exon 14–mutant allele,^3^ *HGF* amplification^44^ and feedback activation of alternate pathways. A number of *MET* resistance mutations, such as D1228H/N, Y1230C/S/H, H1094Y, G1163R, L1195V, F1200I, and R1336W, have been reported.^45–52^ The off-target mechanisms of resistance to c-Metis include *KRAS* amplification and mutation; amplification of *HER3, EGFR, BRAF*, and *KIT;* and NF1 frameshift mutation.^53–57^ Notably, the tumor cell plasticity process known as EMT has been shown to be the prominent nonmutational resistance mechanism for kinase-targeted anticancer therapy.^15,16^ However, the influence of EMT on the response to c-Metis has not been reported in c-Meti-treated NSCLC patients with *MET* dysregulation.

EMT is a dynamic and reversible process, and reversal of EMT may sensitize tumor cells to treatment, leading to a more durable response.^15–17^ Therefore, key regulators that control EMT-related plasticity have the potential to determine the response to anticancer therapy. For example, AXL kinase is associated with mesenchymal features in NSCLC and has been identified as a target to overcome EGFRi resistance,^24^ and in cancer models of acquired resistance to erlotinib (EGFRi), upregulation of AXL correlates with the emergence of EMT features, which has been shown to underlie erlotinib resistance. Both AXL inhibition and knockdown restored sensitivity to erlotinib.^58^ These findings encouraged the ongoing clinical exploration of combination therapy with AXL inhibitors and EGFRis.^59^ In the present study, we focused on the therapy sensitivity-associated epithelial cell state. SYK itself was reported as part of the epithelial core signature,^28,29^ and here, by integrating epithelial cell state scoring with comprehensive omics analysis, SYK was found to regulate the epithelial cell state, dictating sensitivity to c-Metis in c-Met-dysregulated preclinical cancer models and a patient cohort. Furthermore, we revealed the importance of the TGFβ1-Smad2/3-SYK-FRA1 mechanistic axis in epithelial-mesenchymal state regulation and its corresponding role in the response to c-Metis. Decreased SYK expression with shRNA or TGF-β1 treatment accompanied with increasing FRA1 expression, was associated with an enhanced mesenchymal state and decreased sensitivity of cancer cells to c-Metis. In contrast, FRA1 knockdown reversed the mesenchymal cell state and the reduction in inhibitor sensitivity induced by SYK depletion. In addition to downstream canonical Smad2/3 signaling, TGF-β1 can induce a mesenchymal state through noncanonical β-catenin signaling.^60,61^ Sporadic preliminary preclinical studies showed upregulation of β-catenin in c-Meti-resistant NSCLC cells, and β-catenin siRNA increase c-Meti sensitivity.^62^ Our findings together with other reports in cancers with c-Met alterations strongly indicated that the above canonical or noncanonical pathways that could enhance the mesenchymal cell state may be potential therapeutic targets, and intervening in these pathways has the potential to increase drug sensitivity in patients with c-Met alterations who did not respond to c-Meti therapy, which needs further clinical exploration. It appears that comprehensive analysis to identify key regulators of cancer cell epithelial-mesenchymal plasticity may reveal markers related to mesenchymal or epithelial dependency that indicate therapeutic vulnerability and could serve as targets to overcome resistance or as biomarkers for predicting the treatment response; these markers would have important implications in the precise application of anticancer drugs, particularly kinase-targeted therapies. Further extensive exploration may help test this hypothesis to guide anticancer therapy.

Our research shows that SYK has important translational significance and application potential in kinase-targeted therapy.

First, we revealed that “c-Met^+^/SYK^+^”, as a new stratification biomarker, could substantially expand the population of patients predicted to benefit from c-Meti treatment. Our PDX-based parallel trial showed that high SYK expression predicted a therapeutic response to c-Metis in the context of c-Met overexpression, regardless of *MET* gene status (amplification or wild type).

Notably, c-Met overexpression is found in approximately 20 to 25% of NSCLC cases.^3,13,14^ It is usually assessed by IHC with the SP44 antibody and is scored from 0 to 3+ using a semiquantitative approach. There is no agreed-upon cutoff for defining high c-Met expression. In some studies, c-Met staining with moderate (2+) or strong (3+) intensity in ≥50% of tumor cells was used.^3,63^ Thus far, c-Met overexpression by IHC has not been determined to be an effective clinical biomarker with a clear cutoff. The reported response rate in this subpopulation is consistently very low.^11,12^ Intriguingly, in the present study, c-Met overexpression (defined as ≥50% of tumor cells with strong 3+ staining intensity) coupled with SYK overexpression identified a subpopulation of NSCLC patients with a high response rate (73.3%) to c-Metis. This subset of patients also tended to experience a longer PFS time than other patient subsets.

We thus suggest that both SYK and c-Met could be assessed for c-Meti response prediction, as populations overexpressing both SYK and c-Met (c-Met + /SYK + ) are likely to be responsive to c-Meti treatment (Fig. 6b). Moreover, c-Met + /SYK + status is easy to detect by IHC in the clinical setting and has the potential to optimize the current biomarker testing strategy for c-Met-targeted therapy.

Second, SYK has the potential to be a generalizable and sensitive biomarker for other kinase inhibitors in the context of kinase-addicted tumors. We utilized database analysis, cancer cell assays and PDX models and revealed that SYK stratification consistently enriched the population of patients predicted to be responsive to EGFRis and ALKis among those with *EGFR*-mutant and *ALK*-translocated NSCLC, respectively. Similar enrichment was further validated in a cohort of advanced NSCLC patients harboring *EGFR* mutations. When substratified by SYK-high status, all *EGFR*-mutant-positive NSCLC patients responded to EGFRis (ORR, 100%), and their response rate was much higher than the response rate of patients stratified by *EGFR* mutation alone (ORR, 74.2%). The SYK-plus strategy may expand the population of patients with kinase alterations predicted to benefit from kinase inhibitor treatment.

In addition, in the c-Met-dysregulated NSCLC cohort, the response to c-Meti in the SYK^low^ subtype was inconsistent; there was one case of PD (indicating resistance) and 4 cases of SD (indicating clinical benefit) in this cohort. Further stratification by TGFβ1 expression revealed resistance (PD) in the TGFβ1^high^ group (Fig. 5a, c). In Fig. 1c (right panel), the analysis of the EGFRi sensitivity of EGFR-mutant cells using the CCLE database also showed a consistent trend. Cell lines with SYK^low^ expression (5 cell lines, including resistant and sensitive cells) were further stratified based on TGFβ1 expression, and all the cell lines in the SYK^low^/TGFβ1^high^ group exhibited resistance (Fig. 3c). These results indicated that SYK^low^ status combined with TGFβ1^high^ status is more precise in predicting resistance than SYK^low^ status alone. However, in the data from the PDX models (Figs. 4a, 4b), low expression of SYK indicated resistance, and high TGFβ1 expression was not needed to identify the resistant population. We speculated that the number of SYK^low^ PDX models was too small, with only two models for each molecular alteration subtype, which may largely limit the reliability of SYK^low^ alone in predicting resistance in the referenced PDX models. Our study is limited by the relatively small size of the cohorts. The translation potential of the study will be clarified by studies in large cohorts and with the clear definition of a standard cutoff value for IHC staining of c-Met, SYK and TGFβ1 for identifying either sensitivity or resistance. SCC244 (glumetinib), a highly selective c-Met inhibitor, was granted breakthrough therapy designation by the China National Medical Products Administration (NMPA) for the treatment of patients with *MET ex14Δ*-positive metastatic or advanced NSCLC in 2021 (No. CXHB1600071) and orphan drug designation by the FDA for treatment for NSCLC with *MET* genomic aberration in 2022 (https://www.accessdata.fda.gov/scripts/opdlisting/oopd/detailedIndex.cfm?cfgridkey=858221). The New Drug Application (NDA) for SCC244 was submitted to the NMPA in 2022. We hope our ongoing clinical study of SCC244 will help standardize the use of IHC staining and refine reference ranges for clinical or mechanistic studies.

In addition, in the mechanism exploration, we confirmed that Smad2/3-mediated downregulation of SYK, which triggers the resistance to c-Metis, is TGF-β1 dependent. However, in our case, we also found that loss of Smad2/3 enhanced the basal level of SYK without TGF-β1 stimulation only in NCI-H1993 cells, which suggested that Smad2/3 might regulate SYK independent of TGF-β1. Some previous reports have provided similar results. For example, advanced glycation end products (AGEs) could activate Smad2/3 early phosphorylation through ERK/p38.^64^ And STMN-2, markedly up-regulated in HCCs, could activate Smad2 independent of TGF-β1 stimulation.^65^ Therefore, whether TGF-β1-independent Smad regulator is involved in Smad-downregulated SYK in NSCLC needs further exploration.

Furthermore, in the present study, we mainly focused on *MET* and *EGFR* alterations, which are high-frequency typical genetic alterations in lung adenocarcinoma (LUAD) and are rarely detected in lung squamous cell carcinoma (LUSC).^66^ The molecular genomic alterations are quite different between LUAD and LUSC,^66^ the predominantly histologic subtypes of NSCLC. Whether SYK expression is associated with the response to inhibitors of other targetable alterations, including typical LUSC alterations, and the underlying mechanism need to be further studied.

## Materials and Methods

### Omic datasets and analysis

We obtained gene expression, protein expression and drug sensitivity data from CCLE_DepMap_18Q4_RNAseq_log2 (TPM + 1), CCLE_RPPA_20180123.csv, and CCLE_NP24.2009_Drug_data_2015.02.24, respectively, from the CCLE portal (http://www.broadinstitute.org/ccle). Lung cancer cell lines with *MET* amplification, *EGFR* mutation and *ALK* translocation were selected. The protein expression and drug sensitivity data for NCI-H3122 cells were not available on the CCLE. NCI-H3122 is a known ALKi-sensitive cancer cell line; therefore, it was classified as inhibitor-sensitive. Transcriptomic profiling of significantly differentially expressed genes between cancer cell lines considered sensitive versus resistant to specific kinase inhibitors was performed with R using the edgeR package.

### Cell lines

PC-9 cells were purchased from the European Collection of Authenticated Cell Cultures (ECACC). NCI-H3255 cells were purchased from CoBioer (China). NCI-H3122 cells were purchased from the National Cancer Institute (NCI). SPC-A1 cells were purchased from the Cell Bank of the Chinese Academy of Sciences (China). MKN45 and EBC-1 cells were purchased from the Japanese Collection of Research Biosources Cell Bank (JCRB). EBC-1/SR is a cell line with induced acquired resistance to the c-Met selective inhibitor SGX523; it was generated in our lab from the EBC-1 parental cell line by exposure to increasing concentrations of SGX523 for 6 months^37^ HCC827 and HCC827/GR6 cells were kindly provided by Pasi A. Jänne (Dana-Farber Cancer Institute). As described in his previous study,^67^ to generate cell clones resistant to the EGFR inhibitor gefitinib, parental HCC827 cells were exposed to increasing concentrations of gefitinib for 6 months. Six clones isolated from single cells were resistant to gefitinib, one of which was HCC827/GR6. Other cell lines were purchased from the American Type Culture Collection (Manassas, VA, USA). All cell lines were cultured as instructed and were authenticated via short tandem repeats (STR) analysis with the latest test in November 2021 (Genesky Biotechnologies, China) or single-nucleotide polymorphism (SNP) analysis with the latest test in 2022 (Crown Bioscience, China). All cells were routinely tested for mycoplasma and found to be free of contamination. Detailed information on these cell lines is listed in Supplementary Table S2.

### Animal studies

#### Cell line-derived xenograft (CDX) model

PC9-nc/shSYK cells (1 × 10^7^ cells per mouse), EBC-1-nc/shSYK cells (5 × 10^6^ cells per mouse), or MKN45-nc/shSYK cells (5 × 10^6^ cells per mouse) were subcutaneously injected into the right flanks of 5-week-old immunocompromised female BALB/c nude mice. Tumor volume was measured twice per week with a caliper in most tumor models, except for MKN45-nc model which was measured every two days, and quantified by the modified ellipsoidal formula (tumor volume = ½ (length × width^2^)). The mice were randomly assigned to groups when the mean tumor volume reached approximately 100–250 mm^3^ and then given the indicated treatment as described below once daily for a period of time. For the PC9 xenograft model experiment, gefitinib (5 or 10 mg per kg) was suspended in 0.5% sodium carboxymethylcellulose (CMC-Na) and administered daily by oral gavage. Mice in the vehicle groups were treated on the same schedule but were only treated with 0.5% CMC-Na. For the EBC-1 and MKN45 xenograft model experiments, INCB28060 (1 or 3 mg per kg) was suspended in 95% 0.5% methyl cellulose (0.5% MC) and 5% dimethylacetamide (DMAC) and administered daily by oral gavage. Mice in the vehicle groups were treated on the same schedule but were only treated with 95% MC and 5% DMAC.

#### PDX model

Animal studies using NSCLC PDX models were conducted by WuXi AppTec (Shanghai, China) (YM-01-0539, YM-01-0055, YM-01-0251, YM-01-0752, YM-01-0055R, YM-01-0439, YM-01-0555 and YM-01-0538; *n* = 6 in each group for YM-01-0055R; *n* = 8 for the remaining models) and Crown Bioscience (LU1235, LU5251, LU1901, LU0858, LU2503, LU2071, LU6412, and LU6425; *n* = 8 per control group and *n* = 6 per experimental group for LU1901 and LU0858; *n* = 8 for the remaining models). The mice were given vehicle alone or the indicated compound via oral gavage once daily for 13–35 days. Tumor size was measured twice per week using a microcaliper.

#### Study approval

Animal studies using CDX models were approved by the Institutional Animal Care and Use Committee of the Shanghai Institute of Materia Medica (approval numbers 2017-04-DJ-26 and 2022-06-DJ-70). Animal studies using PDX models were approved by the Institutional Animal Care and Use Committees of WuXi AppTec (approval N20160615-mouse and approval ON01-003-2017 V1.1) and Crown Bioscience (approval numbers AN-1507-011-341 and EB17-30).

### Human subject study

#### EGFR cohort

Thirty-one patients with advanced NSCLC harboring activating *EGFR* mutations were included. Patients with simultaneous T790M mutations were excluded. The patients were 76 years old or younger, and all had measurable disease. Approval was obtained from institutional research ethics boards at the following institutions: Renji Hospital, School of Medicine, Shanghai Jiao Tong University; the Affiliated Hospital of Xuzhou Medical University; and the Central Hospital of Minhang District. Informed consent for biomarker analysis was obtained from each patient. *EGFR* mutations were detected using amplification refractory mutation system (ARMS) real-time PCR analysis with tumor tissue obtained via bronchoscopy, percutaneous lung biopsy, or surgical resection. The clinical parameters of all patients, including sex, age, smoking status, pathomorphological diagnosis, disease stage, *EGFR* gene mutation type, and prior treatment, such as surgery or chemotherapy, were collected. All patients received first-generation kinase inhibitors (gefitinib at a dosage of 250 mg daily orally, erlotinib at a dosage of 150 mg daily orally, and icotinib at a dosage of 125 mg three times daily orally).

#### *MET* cohort

Twenty patients with advanced NSCLC who had c-Met protein overexpression and were treated with crizotinib or capmatinib were included (Supplementary Table S6). Patients with *EGFR* mutations, *ROS1* rearrangements or *ALK* rearrangements were excluded. All patients had measurable disease. Research approval was obtained from the institutional research ethics board at Guangdong Provincial People’s Hospital. Informed consent for the biomarker analysis was obtained from each patient. *MET* gene amplification was assessed by fluorescence in situ hybridization (FISH), and c-Met protein overexpression was assessed by IHC with an SP44 antibody, as previously described.^11^ *MET* amplification was defined as either *MET* gene copy number (GCN) ≥ 6 and/or a *MET*/centromere ratio of ≥2.0. c-Met overexpression was defined as ≥50% of tumor cells with strong staining intensity (3+). Clinical parameters for all patients, including sex, age, smoking status, histologic diagnosis, disease stage, and prior treatment information, were collected. All patients received the c-Meti crizotinib at a dosage of 150 mg daily orally.

Clinical assessment was performed based on RECIST, version 1.1. Treatment response was determined by an external review of the CT films by experienced experts.

The details for representative cases in the EGFR group and MET group are shown in the Supporting Methods section.

Tumor tissues obtained before treatment were analyzed by IHC. Staining was assessed by two independent pathologists with no prior knowledge of patient characteristics. Discrepancies were resolved by consensus. Semiquantitative analysis of SYK and TGF-β1 immunoreactivity was performed using the H-score system. The percentage of immunoreactive tumor cells was assessed on a scale of 0~3 (0: 0~10%, 1: 11~45%, 2: 46~75%, and 3: 76~100%). Staining intensity was scored on a scale of 0~2 (weak: 0, moderate: 1, and strong: 2). The H-score included scores of 0~4 and 6 and was calculated by multiplying the staining percentage score by the intensity score, resulting in a negative (0, 1, and 2) or a positive (3, 4, and 6) expression value for SYK and a negative (0, 1, 2, and 3) or a positive (4 and 6) expression value for TGF-β1. The antibodies used for IHC were anti-TGF-β1 antibody (ab92486) (Abcam, Cambridge, MA, USA) and anti-SYK antibody (4D10) (sc-1240) (Santa Cruz, CA, USA).

### Statistical analysis

The data are presented as the mean ± SD for in vitro data or the mean ± SEM for in vivo data. Data were analyzed using unpaired two-sided t tests (two groups) or one-way ANOVA (≥3 groups) using GraphPad Prism software. Pearson’s correlation was used to analyze the relationships between the indicated groups. Pearson’s chi-square and Fisher’s exact tests were used for comparative analysis using SPSS version 23. Statistical significance was determined based on the P value: not significant (NS), *p* > 0.05; *p < 0.05; **p < 0.01; and ***p < 0.001.

## Supplementary information

Supplementary manuscript

## Data Availability

The data needed to evaluate the conclusions in this study are available within the article and/or its supplementary data files. The RNA-sequencing data generated in this study are publicly available in Gene Expression Omnibus (GEO) dataset GSE125956. Other data are available upon reasonable request from the corresponding author. Extended materials and methods are provided in the Supplementary materials and methods.
