# Supplementary material for: SYK-mediated epithelial cell state is associated with response to c-Met inhibitors in c-Met-overexpressing lung cancer
Source: Signal Transduct Target Ther. 2023 May 15;8:185. doi: 10.1038/s41392-023-01403-w (PMC10183461; doi:10.1038/s41392-023-01403-w)
Supplement: Supplementary file 1 — Supplementary manuscript [file 41392_2023_1403_MOESM1_ESM.docx]

Supplementary Materials for

SYK-mediated Epithelial Cell State is Associated with Response to c-Met Inhibitors in c-Met-overexpressing Lung Cancer

Ji Zhou^1,5,§^, Xu-Chao Zhang^2,§^, Shan Xue^3,§^, Mengdi Dai^1,5,§^, Yueliang Wang^1^, Xia Peng^1^, Jianjiao Chen^8^, Xinyi Wang^1^, Yanyan Shen^1^, Hui Qin^3^, Bi Chen^6^, Yu Zheng^3^, Xiwen Gao^7^, Zuoquan Xie^1,5^, Jian Ding^1,4,5^, Handong Jiang^3,*^, Yi-Long Wu^2,*^, Meiyu Geng^1,4,5,*^, Jing Ai^1,4,5,*^

Correspondence to: Handong Jiang( [jianghd@163.com](mailto:jianghd@163.com)), Yi-Long Wu ([syylwu@live.cn](mailto:syylwu@live.cn)), Meiyu Geng ([mygeng@simm.ac.cn](mailto:mygeng@simm.ac.cn)), Jing Ai (jai@simm.ac.cn).

**This PDF file includes:**

Supplementary Materials and Methods

References

Tables S1 to S7

Figures. S1 to S16

Supplementary Materials and Methods

Gene set enrichment analysis (GSEA)

GSEA^1^ was performed based on RNA-seq data from triplicates with permutation analysis, using software from the Broad Institute (www.broadinstitute.org/gsea), with 1000 phenotype permutations and default values for other parameters. The gene sets used in this study were from four cell- or patient-derived signatures of a highly epithelial state and a highly mesenchymal state defined by Taube^2^, Groger^3^, Mak^4^ and Byers^5^.

Computation of epithelial and mesenchymal (E/M) scores

To compute the E/M scores of a sample, we performed gene set variation analysis (GSVA) with the Bioconductor R package gsva^6^ (Supplementary Table S1). The gene sets used were the same as those used for GSEA. We used log-transformed RNA-seq transcripts per million (TPM) data from 14 lung cell lines, which were extracted from DepMap Q4, as the expression input for GSVA. The output was the enrichment score of the epithelial and mesenchymal gene sets.

Gene Ontology (GO) enrichment analysis

Four mesenchymal gene lists^2-5^ were evaluated by GO analysis coupled with the functional annotation tool of the Database for Annotation, Visualization and Integrated Discovery (DAVID) v6.8. Significant gene sets in the molecular function, biological process and cell component categories are shown according to the number of enriched genes, with a threshold cutoff of p < 0.05 and false discovery rate (FDR) <0.05. Byers' mesenchymal signature (M-sig) also showed enrichment in the extracellular matrix (data not shown), although the FDR was higher than 0.05.

RNA sequencing (RNA-seq)

RNA-seq was performed by Novogene. RNA was extracted using TRIzol reagent (Thermo Fisher) and analyzed using an Agilent 2100 Bioanalyzer. Sequencing was performed using an Illumina HiSeq system. The sequences were aligned to the human genome (hg38), and GSEA was performed using GSEA software. The four E/M signature gene sets were used to assess the enrichment of E/M signature-associated genes in the treatment group versus the vehicle group.

Compounds and reagents

SCC244 (glumetinib), a novel selective c-Meti^7^, was obtained from Haihe Biopharma (Shanghai, China). Gefitinib, capmatinib and LY2157299 were purchased from Melone Pharma (Dalian, China). Puromycin was purchased from InvivoGen (San Diego, USA). Sulforhodamine B (SRB) sodium salt and hexadimethrine bromide (polybrene) were purchased from Sigma‒Aldrich (St. Louis, MO, USA). Other reagents were purchased from Selleck Chemicals (Houston, USA).

Antibodies

Antibodies specific for SYK (4D10) (sc-1240, 1:500), c-Met (C-28) (sc-161, 1:500), FRA1 (C-12) (sc-28310, 1:1000) and N-cadherin (8C11) (sc-53488, 1:500) were purchased from Santa Cruz Biotechnology (Santa Cruz, CA, USA). Antibodies specific for phospho-EGF receptor (Tyr1068) (No. 2234, 1:1000), EGF receptor (D38B1) (No. 4267, 1:1000), phospho-c-Met (Tyr1234/1235) (D26) (No. 3077, 1:1000), phospho-ALK (Tyr1278) (D59G10) (No. 6941, 1:1000), ALK (D5F3) (No. 3633, 1:1000), phospho-Akt (Ser473) (D9E) (No. 4060, 1:1000), Akt (pan) (C67E7) (No. 4691, 1:1000), phospho-p44/42 MAPK (Erk1/2) (Thr202/Tyr204) (No. 9101, 1:5000), p44/42 MAPK (Erk1/2) (137F5) (No. 4695, 1:2000), phospho-Smad2 (Ser465/467) (138D4) (No. 3108, 1:1000), Smad2 (D43B4) (No. 5339, 1:1000), phospho-Smad3 (Ser423/425) (C25A9) (No. 9520, 1:1000), Smad3 (C67H9) (No. 9523, 1:1000), TGF-β1 (No. 3711, 1:1000), Slug (C19G7) (No. 9585, 1:1000), β-tubulin (No. 2146, 1:2000) and Sp1 (D4C3) (No. 9389, 1:1000) were purchased from Cell Signaling Technologies (Beverly, MA, USA). Antibodies specific for vimentin [V9] (ab8069, 1:2000) and fibronectin [IST-9] (ab6328, 1:1000) were purchased from Abcam (Cambridge, MA, USA). An anti-phospho-SYK (Y525/Y526) antibody (MAB6459, 1:1000) was purchased from R&D Systems. A purified mouse anti-E-cadherin (610181, 1:2000) antibody was purchased from BD Biosciences (San Diego, California). An anti-ZEB1 antibody (NBP1-05987, 1:1000) was purchased from Novus Biologicals (Littleton, Colorado, USA). An anti-β-actin antibody (AM1021B, 1:5000) was purchased from Abgent (San Diego, CA, USA). A monoclonal mouse antibody specific for glyceraldehyde 3-phosphate dehydrogenase (GAPDH) (KC-5G4, 1:10,000) was purchased from KANGCHEN (Shanghai, China).

The secondary antibodies used in this study were peroxidase-conjugated AffiniPure goat anti-rabbit IgG (H+L) (No. 111-035-003) and goat anti-mouse IgG (H+L) (No. 115-035-003), which were obtained from Jackson ImmunoResearch (Newmarket, UK). The secondary antibodies were used at a 1:2000 dilution.

Coimmunoprecipitation (co-IP) assay

The co-IP assay was performed as follows: cells were lysed in IP lysis buffer (No. P0013, Beyotime, Shanghai, China) containing protease inhibitors and phosphatase inhibitors (Roche Applied Science, Mannheim, Germany) on ice for 1 h. Then, the crude lysates were centrifuged at 14,000 x g for 15 min at 4 °C, and the supernatants were collected. After protein quantification, supernatants were incubated with appropriate antibodies (SYK (4D10) (sc-1240) and Sp1 (D4C3) (No. 9389)) with rocking overnight at 4 °C. Protein A/G magnetic beads (YJ003, Epizyme, China) were added, followed by gentle rocking for 6 h at 4 °C. The next day, the beads were washed three times with cold lysis buffer. Proteins were eluted by boiling in 1×SDS lysis buffer and analyzed by western blotting.

Gene knockdown

An shRNA targeting SYK (shSYK, TRCN0000003166) was engineered and packaged using a lentiviral delivery system. The lentivirus that expressed the shRNA was produced using 293T cells with the packaging systems psPAX2 and pMD2.G. siRNAs targeting Smad2 and Smad3 were administered using Lipofectamine RNAiMax (#13778500) according to the manufacturer’s instructions. The siRNA sequences were as follows: Smad2&3 siRNA-1: 5’-GUGGUUGGACAAAGUAUUA-3’, 5’-GGGCGUAUGUAACAUUAGU-3’; Smad2&3 siRNA-2: 5’-GGUGUUCGAUAGCAUAUUA-3’, 5’-GCCUGGUCAAGAAACUCAA-3’; Sp1 siRNA-1: 5’- GCCGUUGGCUAUAGCAAAUTT-3’; Sp1 siRNA-2: 5’-GCCCUUAUUACCACCAAUATT-3’; FRA1 siRNA-1: 5’-GAGGGCAGCUGCUAUUUAUTT-3’; FRA1 siRNA-2: 5’- GCCCAGAGACUUUGUAGAUTT-3’.

Cell proliferation assay

Cells were seeded in 96-well culture plates (Corning, USA), incubated overnight and treated with the indicated drugs for 72 h. Finally, cell proliferation was determined using SRB or Cell Counting Kit-8 (CCK-8) assays (Vazyme, USA). The half-maximal inhibitory concentration (IC_50_) values were calculated by concentration‒response curve fitting using the four-parameter method. Error bars represent the mean ± standard deviation (SD) from two to four independent experiments.

Migration assay

Negative control HCC827 (HCC827-nc) cells or SYK shRNA-transfected HCC827 (HCC827-shSYK) cells were resuspended in 100 μL of RPMI 1640 control medium and placed in the upper well of a Transwell system. A migration assay was performed by adding 600 μL of RPMI 1640 control medium supplemented with 10% fetal bovine serum (FBS) to the bottom well. The cells were then allowed to migrate to the bottom of the chamber for 24 h. The number of transmigrated HCC827-shSYK cells relative to that of HCC827-nc cells was then measured.

Annexin V apoptosis assay

Cells were collected and analyzed for annexin V and propidium iodide (PI) staining according to the manufacturer’s protocol (BD Biosciences, USA). Briefly, the cells were seeded at an appropriate density in 6-well plates (Corning, USA) and treated with the indicated reagents. After 48 h, the cells and supernatants were collected, washed with phosphate-buffered saline (PBS), and stained with annexin V and PI for 15 min. The samples were then analyzed with a FACSCalibur flow cytometer (BD, USA).

Cell cycle arrest assay

Cells were collected and analyzed for propidium iodide (PI) staining according to the manufacturer’s protocol (BD Biosciences, USA). Briefly, the cells were seeded at an appropriate density in 6-well plates (Corning, USA). After FRA1 siRNA transfection for 24 h, cells were treated with the indicated reagents for another 24 h. After that, cells and supernatants were collected, washed with phosphate-buffered saline (PBS), and then fixed in ice-old 70% ethanol added dropwise overnight at -20 °C. After further washes with PBS, cells were stained with propidium iodide/RNase staining buffer (550825, BD Pharmingen) at room temperature for 30 min and immediately analyzed using flow cytometry.

Quantitative real-time PCR

Total RNA was isolated from cells using the MiniBEST RNA Extraction Kit (Takara, Japan). cDNA was synthesized with the PrimeScript RT Reagent Kit (Takara, Japan) and then subjected to PCR using SYBR Premix Ex Taq (Takara, Japan) on an ABI VAII7 system (Thermo, USA). The amplification results were analyzed with QuantStudio software (Thermo, USA). All primers for qRT‒PCR are described in Supplementary Table S6. All assays were replicated three times.

Western blot analysis

Protein lysates from tissues or cells were obtained using preheated 2% sodium dodecyl sulfate (SDS) and analyzed by Western blot analysis, as described previously^8^. The protein expression levels were quantified with Image Lab software and normalized to the levels of β-actin or GAPDH.

Detection of TGF-β1 and SYK

Cancer cell lines were plated in 6-well plates (Corning, USA). After culturing the cells for 48 h, the supernatants were collected and centrifuged at a speed of 12,000 × g for 5 min to remove cell debris. Then, the concentration of TGF-β1 was assessed using the Quantikine Human TGF-β1 ELISA Kit (R&D, USA). The SYK and TGF-β1 mRNA levels in the cells were assessed by real-time PCR analysis and were normalized to those of β-actin. SYK protein expression was measured by immunoblotting and semiquantified by densitometric analysis.

For the *in vivo* models, SYK and TGF-β1 protein levels were measured by immunoblotting and semiquantified by densitometric analysis.

Chromatin immunoprecipitation (ChIP) assays

ChIP assays were performed using the ChIP-IT Express Kit (Active Motif, 53008) according to the procedures provided by the manufacturer. The antibodies for ChIP were as follows: anti-Smad2/3 (D7G7) XP rabbit monoclonal antibody (mAb) (CST, 8685), normal rabbit IgG (CST, 2729), and anti-histone H3 (D2B12) XP rabbit mAb (CST, 4620). The resulting DNA was analyzed by real-time PCR using primers that encompassed the smad2/3 binding site in the SYK promoter, which was predicted by PROMO (http://alggen.lsi.upc.es/cgi-bin/promo_v3/promo/promoinit.cgi? dirDB=TF_8.3). Error bars represent the mean ± SD from three independent experiments. The following primers were used: forward, 5'-TGGAGCTTGTTGGTTTGGTG-3' and reverse, 5'-CGGTGGCTTCTGTGACTGC-3'. The PCR products were detected with 3% TBE agarose gel analysis.

The details for representative cases in the EGFR cohort and MET group cohort.

The details for representative cases in the *EGFR* group are as follows. Case ELU-18 involved a 67-year-old female patient with advanced NSCLC harboring the *EGFR* L858R mutation who was treated with gefitinib. The CT images after 3 months of treatment showed a significant decrease in tumor size and the disappearance of most pulmonary metastases. Case ELU-12 involved a 48-year-old male patient with advanced NSCLC harboring the *EGFR* exon 19 del mutation who was treated with erlotinib. The CT images after 3 months of treatment showed a significant decrease in tumor size and the disappearance of most pulmonary metastases. Case ELU-10 involved a 62-year-old female patient with advanced NSCLC harboring the *EGFR* L858R mutation who was treated with gefitinib. The CT images after 4.5 months of treatment showed no obvious changes in tumor size.

The details for representative cases in the *MET* group are as follows. Case MLU-12 involved a 59-year-old male patient who was a 30 pack-year smoker with advanced pulmonary adenocarcinoma exhibiting *de novo* c-Met overexpression (IHC 100%, +++), high expression of SYK and low expression of TGF-β1. After first-line crizotinib treatment, a CT scan showed a significant partial response (PR). The patient achieved a PFS time of 7.0 months. Case MLU-09 involved a 64-year-old male patient who was a 30 pack-year smoker with advanced pulmonary adenocarcinoma exhibiting *de novo* c-Met overexpression (IHC 100%, +++), high expression of SYK and low expression of TGF-β1. After first-line crizotinib treatment, the patient exhibited a PR and achieved a PFS of 8.1 months. Case MLU-02 involved a 72-year-old male patient who was a 100 pack-year smoker with advanced pulmonary adenocarcinoma exhibiting *de novo* c-Met overexpression (IHC 100%, +++), high expression of SYK and low expression of TGF-β1. After first-line crizotinib treatment, the patient exhibited a PR and achieved a PFS of 16.2 months. At disease progression based on assessment by CT scan, IHC showed that compared to baseline specimens, tumor specimens after progression exhibited reduced SYK expression and increased TGF-β1 expression.

Analysis of E-cadherin and SYK expression association

A total of 102 NSCLC specimens were analyzed by IHC. The percentage of immunoreactive tumor cells was on a scale of 0-3, corresponding to the percentage of immunoreactive tumor cells (0%-10%, 11%-45%, 46%-75% and 75%-100%, respectively). The staining intensity was scored as negative or weak (score = 0), moderate (score = 1), or strong (score = 2). A score ranging from 0~6 was calculated by multiplying the staining percentage score with the intensity score, resulting in a negative (0-2) or a positive (3, 4, and 6) expression value for SYK and a low (0-4) or a high (6) expression value for E-cadherin. Data were analyzed by crosstab analysis using the Pearson chi-square test.

References

1 Subramanian, A. *et al.* Gene set enrichment analysis: a knowledge-based approach for interpreting genome-wide expression profiles. *Proc Natl Acad Sci U S A* **102**, 15545-15550 (2005).

2 Taube, J. H. *et al.* Core epithelial-to-mesenchymal transition interactome gene-expression signature is associated with claudin-low and metaplastic breast cancer subtypes. *Proc Natl Acad Sci U S A* **107**, 15449-15454 (2010).

3 Gröger, C. J., Grubinger, M., Waldhör, T., Vierlinger, K. & Mikulits, W. Meta-analysis of gene expression signatures defining the epithelial to mesenchymal transition during cancer progression. *PLoS One* **7**, e51136 (2012).

4 Mak, M. P. *et al.* A Patient-Derived, Pan-Cancer EMT Signature Identifies Global Molecular Alterations and Immune Target Enrichment Following Epithelial-to-Mesenchymal Transition. *Clin Cancer Res* **22**, 609-620 (2016).

5 Byers, L. A. *et al.* An epithelial-mesenchymal transition gene signature predicts resistance to EGFR and PI3K inhibitors and identifies Axl as a therapeutic target for overcoming EGFR inhibitor resistance. *Clin Cancer Res* **19**, 279-290 (2013).

6 Hänzelmann, S., Castelo, R. & Guinney, J. GSVA: gene set variation analysis for microarray and RNA-seq data. *BMC Bioinformatics* **14**, 7 (2013).

7 Ai, J. *et al.* Preclinical Evaluation of SCC244 (Glumetinib), a Novel, Potent, and Highly Selective Inhibitor of c-Met in MET-dependent Cancer Models. *Mol Cancer Ther* **17**, 751-762 (2018).

8 Liu, H. *et al.* c-Myc Alteration Determines the Therapeutic Response to FGFR Inhibitors. *Clin Cancer Res* **23**, 974-984 (2017).

Supplementary Tables

Table S1. E/M scores of 14 cancer cell lines in the CCLE database computed by GSVA.

| Cell Line | Taube | | Groger | | Mak | | Byers | |
| --- | --- | --- | --- | --- | --- | --- | --- | --- |
|  | E_score | M_score | E_score | M_score | E_score | M_score | E_score | M_score |
| HCC2935_LUNG | 0.1934 | -0.0550 | 0.3052 | -0.3399 | 0.4966 | -0.4151 | 0.5901 | -0.3956 |
| HCC4006_LUNG | 0.1593 | 0.3583 | 0.2831 | 0.1566 | 0.1327 | 0.3045 | 0.3763 | 0.1290 |
| NCIH3255_LUNG | 0.2592 | -0.1518 | 0.3448 | -0.2227 | 0.5707 | -0.4035 | 0.6398 | 0.0805 |
| NCIH2228_LUNG | -0.3948 | 0.2173 | -0.4168 | 0.4034 | -0.6579 | 0.3814 | -0.6673 | 0.5252 |
| NCIH3122_LUNG | 0.2665 | -0.3102 | 0.3570 | -0.4741 | 0.0224 | -0.2666 | 0.4999 | -0.2209 |
| PC9_LUNG | 0.4671 | -0.0714 | 0.4415 | -0.0406 | 0.1999 | -0.1776 | 0.4202 | -0.1919 |
| EBC1_LUNG | -0.0794 | -0.3282 | -0.1374 | -0.2883 | 0.2935 | -0.3669 | -0.0104 | -0.0269 |
| HCC827_LUNG | 0.0706 | -0.2993 | -0.0632 | -0.0098 | 0.2664 | 0.1009 | -0.0564 | 0.1333 |
| NCIH1650_LUNG | 0.2242 | 0.1503 | 0.1964 | 0.1011 | 0.5122 | 0.2435 | 0.1643 | -0.3162 |
| NCIH1648_LUNG | 0.4025 | -0.5214 | 0.3994 | -0.4585 | -0.0597 | -0.5407 | 0.4216 | -0.2476 |
| NCIH1573_LUNG | -0.1613 | -0.4810 | 0.0578 | -0.5884 | 0.4193 | -0.5804 | 0.2578 | -0.7321 |
| NCIH1975_LUNG | -0.4623 | 0.1582 | -0.5423 | 0.3374 | -0.6781 | 0.3646 | -0.6473 | 0.4652 |
| LOUNH91_LUNG | -0.5136 | 0.4095 | -0.6412 | 0.5053 | -0.7714 | 0.5353 | -0.7349 | 0.0805 |
| NCIH2172_LUNG | -0.5771 | 0.3005 | -0.6235 | 0.2943 | -0.7882 | 0.3174 | -0.7875 | -0.2384 |

E_score, epithelial signature score; M_score, mesenchymal signature score

Table S2. Detailed information on the cancer cell lines used in this study

| Molecular Classification | Cell lines | Target gene aberration | Cancer Type |
| --- | --- | --- | --- |
| EGFR | NCI-H1650 | Exon 19del | lung adenocarcinoma |
|  | NCI-H1975 | L858R, T790M |  |
|  | NCI-H3255 | L858R |  |
|  | PC-9 | Exon 19del |  |
|  | HCC827 | Exon 19del |  |
|  | HCC827/GR6 | Exon 19del |  |
|  | HCC2935 | Exon 19del |  |
|  | HCC4006 | Exon 19del |  |
| ALK | NCI-H3122 | EML4-ALK | lung adenocarcinoma |
|  | NCI-H2228 |  |  |
| MET | EBC-1 | Amplification | squamous cell lung cancer |
|  | EBC-1/SR |  | squamous cell lung cancer |
|  | NCI-H1993 |  | NSCLC |
|  | MKN45 |  | gastric cancer |
| RET | TT | C634R | thyroid carcinoma |
| KRAS | NCI-H23 | G12C | lung adenocarcinoma |
|  | NCI-H1792 | G12C | lung adenocarcinoma |
|  | NCI-H358 | G12C | lung adenocarcinoma |
|  | NCI-H441 | G12V | lung adenocarcinoma |
|  | A549 | G12S | lung adenocarcinoma |
|  | NCI-H727 | G12V | carcinoid lung cancer |
|  | NCI-H460 | Q61H | large cell lung cancer |
| NRAS | NCI-H1299 | Q61K | NSCLC |
| Others | Calu-3 | ND | lung adenocarcinoma |
|  | NCI-H226 |  | lung adenocarcinoma |
|  | NCI-H522 |  | NSCLC |
|  | NCI-H661 |  | large cell lung cancer |
|  | SPC-A1 |  | lung adenocarcinoma |

Table S3. Genes involved in the extracellular matrix in the mesenchymal gene set defined by Taube, Groger and Mak.

| Geneset | Genes involved in extracellular matrix |
| --- | --- |
| Taube M-sig | LTBP1, LTBP2, TGFB1I1, FBLN1, FBLN5, BGN, DCN, VIM, MMP1, MMP2, COL1A2, COL5A2, LMCD1, DBT, ECM1, FBN1, NID1, POSTN, PCOLCE |
| Groger M-sig | LTBP1, LTBP2, FBLN1, FBLN5, DCN, LUM, VIM, MMP2, SERPINE1, SERPINE2, COL1A1, COL5A1, COL6A3, FBN1, FN1, NID2, PLAT, TGM2, VCAN |
| Mak M-sig | THBS2, LOXL2, VIM, MMP2, COL1A1, COL1A2, COL5A1, COL5A2, COL6A1, COL6A2, COL6A3, COL8A1, FBN1, FN1, ADAMTS12, HTRA1, NID2, POSTN, PCOLCE, VCAN |

Table S4. NSCLC PDX models with EGFR and MET aberrations/c-Met overexpression.

| Molecular Classification | Models | Target gene aberration | Other hotspot mutation | SYK level | Responsiveness |
| --- | --- | --- | --- | --- | --- |
| EGFR | YM-01-0539 | Exon19 del | ND | H | Responder |
|  | YM-01-0055 | L858R | ND | H | Responder |
|  | YM-01-0251 | Exon19 del | ND | H | Responder |
|  | YM-01-0752 | Exon19 del | ND | H | Responder |
|  | LU1235 | Exon19 del | ND | H | Responder |
|  | LU5251 | L858R | ND | H | Responder |
|  | LU1901 | G719A | *MET* Amp | L | Nonresponder |
|  | LU0858 | L858R | *MET* Amp | L | Nonresponder |
|  | YM-01-0055R | L858R | ND | L | Acquired-resistance |
| c-Met | LU2503 | Amp, Exon14 del | ND | H | Responder |
|  | LU2071 | overexpression | ND | H | Responder |
|  | LU6412 | Amp | ND | H | Responder |
|  | LU6425 | Amp | ND | H | Responder |
|  | YM-01-0439 | overexpression | ND | H | Responder |
|  | YM-01-0555 | overexpression | ND | L | Nonresponder |
|  | YM-01-0538 | overexpression | ND | L | Nonresponder |

ND, none-detected; Amp, amplification; del, deletion

The *MET* copy number of the *MET*-amplified PDX models used in this study was greater than 5.

Table S5. Clinical characteristics of the 31 NSCLC patients with EGFR mutations receiving the indicated first-generation EGFRi.

| Sample ID | Sex | *EGFR* Mutation | EGFRi Receiving | Best Response |
| --- | --- | --- | --- | --- |
| ELU-01 | Male | Exon19 del | icotinib | PR |
| ELU-02 | Male | Exon19 del | gefitinib | PR |
| ELU-03 | Female | Exon19 del | icotinib | PR |
| ELU-04 | Female | Exon19 del | gefitinib | PR |
| ELU-05 | Female | G719X | erlotinib | PR |
| ELU-06 | Male | Exon19 del | icotinib | PR |
| ELU-07 | Female | L858R | gefitinib | SD |
| ELU-08 | Female | Exon19 del | icotinib | SD |
| ELU-09 | Female | Exon19 del | gefitinib | SD |
| ELU-10 | Female | L858R | gefitinib | SD |
| ELU-11 | Female | Exon19 del, L858R | icotinib | PR |
| ELU-12 | Male | Exon19 del | erlotinib | PR |
| ELU-13 | Female | L858R | gefitinib | PR |
| ELU-14 | Male | Exon19 del | gefitinib | PR |
| ELU-15 | Male | Exon19 del | gefitinib | PR |
| ELU-16 | Female | L858R | icotinib | PR |
| ELU-17 | Male | L858R | gefitinib | PR |
| ELU-18 | Female | L858R | gefitinib | PR |
| ELU-19 | Female | L858R | gefitinib | PR |
| ELU-20 | Male | Exon19 del | erlotinib | PR |
| ELU-21 | Female | Exon19 del | gefitinib | PR |
| ELU-22 | Female | Exon19 del | icotinib | PR |
| ELU-23 | Female | L858R | gefitinib | PR |
| ELU-24 | Male | L858R | gefitinib | PR |
| ELU-25 | Female | Exon19 del | gefitinib | PR |
| ELU-26 | Male | L858R | icotinib | PR |
| ELU-27 | Female | L861Q | gefitinib | PR |
| ELU-28 | Female | Exon19 del | icotinib | SD |
| ELU-29 | Female | L858R | gefitinib | SD |
| ELU-30 | Female | L858R | gefitinib | SD |
| ELU-31 | Female | L858R | gefitinib | SD |

The treatment was continued until disease progression, unacceptable toxicity, or patient refusal.

Table S6. Clinical characteristics of NSCLC patients receiving c-Met-targeted therapy.

| **Sample ID** | **Sex** | **c-Met IHC** | ***MET* Aberration** | **Treatment** | **Best Response** |
| --- | --- | --- | --- | --- | --- |
| MLU-01 | Male | Positive | Amp | crizotinib | SD |
| MLU-02 | Male | Positive | Amp | crizotinib | PR |
| MLU-03 | Male | Positive | WT | capmatinib | SD |
| MLU-04 | Male | Positive | WT | crizotinib | PR |
| MLU-05 | Female | Positive | WT | capmatinib | PR |
| MLU-06 | Male | Positive | WT | crizotinib | PR |
| MLU-07 | Male | Positive | Amp | crizotinib | PR |
| MLU-08 | Male | Positive | Amp | crizotinib | SD |
| MLU-09 | Male | Positive | Amp | crizotinib | PR |
| MLU-10 | Female | Positive | Amp | crizotinib | PR |
| MLU-11 | Male | Positive | Amp | crizotinib | PR |
| MLU-12 | Male | Positive | Amp | crizotinib | PR |
| MLU-13 | Female | Positive | WT | capmatinib | SD |
| MLU-14 | Male | Positive | WT | crizotinib | SD |
| MLU-15 | Male | Positive | WT | crizotinib | PR |
| MLU-16 | Male | Positive | WT | crizotinib | SD |
| MLU-17 | Male | Positive | WT | crizotinib | PR |
| MLU-18 | Female | Positive | WT | capmatinib | PD |
| MLU-19 | Male | Positive | WT | crizotinib | SD |
| MLU-20 | Male | Positive | Amp | crizotinib | SD |

The treatment was continued until disease progression, unacceptable toxicity, or patient refusal.

Table S7. Primers used in this study.

| Gene | Forward (5’🡪3’) | Reverse (5’🡪3’) |
| --- | --- | --- |
| ACTB | ACAGAGCCTCGCCTTTGCCGAT | AGTTGGTGACGATGCCGTGCT |
| SYK | TGGCAGCTAGTCGAGCAT | CTGACCAAGTCGCAGGAT |
| VIM | TAATCTGGATTCACTCCCTCT | AGTTTCGTTGATAACCTGTCC |
| CDH1 | CTGAGAACGAGGCTAACG | GTCCACCATCATCATTCAATAT |
| CTGF | GACCCAACTATGATTAGAGCCA | GGAGATGCCCATCCCACA |
| CDH2 | AATCGACTTTGAAACAAATAGG | ACTGAGGCGGGTGCTGAA |
| FN1 | CCAGATAACAGGATACCGAC | AGACAGAGGGACCCACAT |
| SNAI1 | CGCTCTTTCCTCGTCAGG | GGCTGCTGGAAGGTAAACTC |
| SNAI2 | CAGCTCAGGAGCATACAG | GAGGAGGTGTCAGATGGA |
| ZEB1 | AAGTGGCGGTAGATGGTA | TGTTGTATGGGTGAAGCA |
| EPCAM | GCCAGTGTACTTCAGTTGG | AGGTTTTGCTCTTCTCCC |
| BIK | GGACGAGATGGACGTGAG | CGTAGATGAAAGCCAGACC |
| SP1 | TGGCAGCAGTACCAATGGC | CCAGGTAGTCCTGTCAGAACTT |
| FOSL1 | CAGGCGGAGACTGACAAACTG | TCCTTCCGGGATTTTGCAGAT |

Supplementary Figures and Figure legends

­­
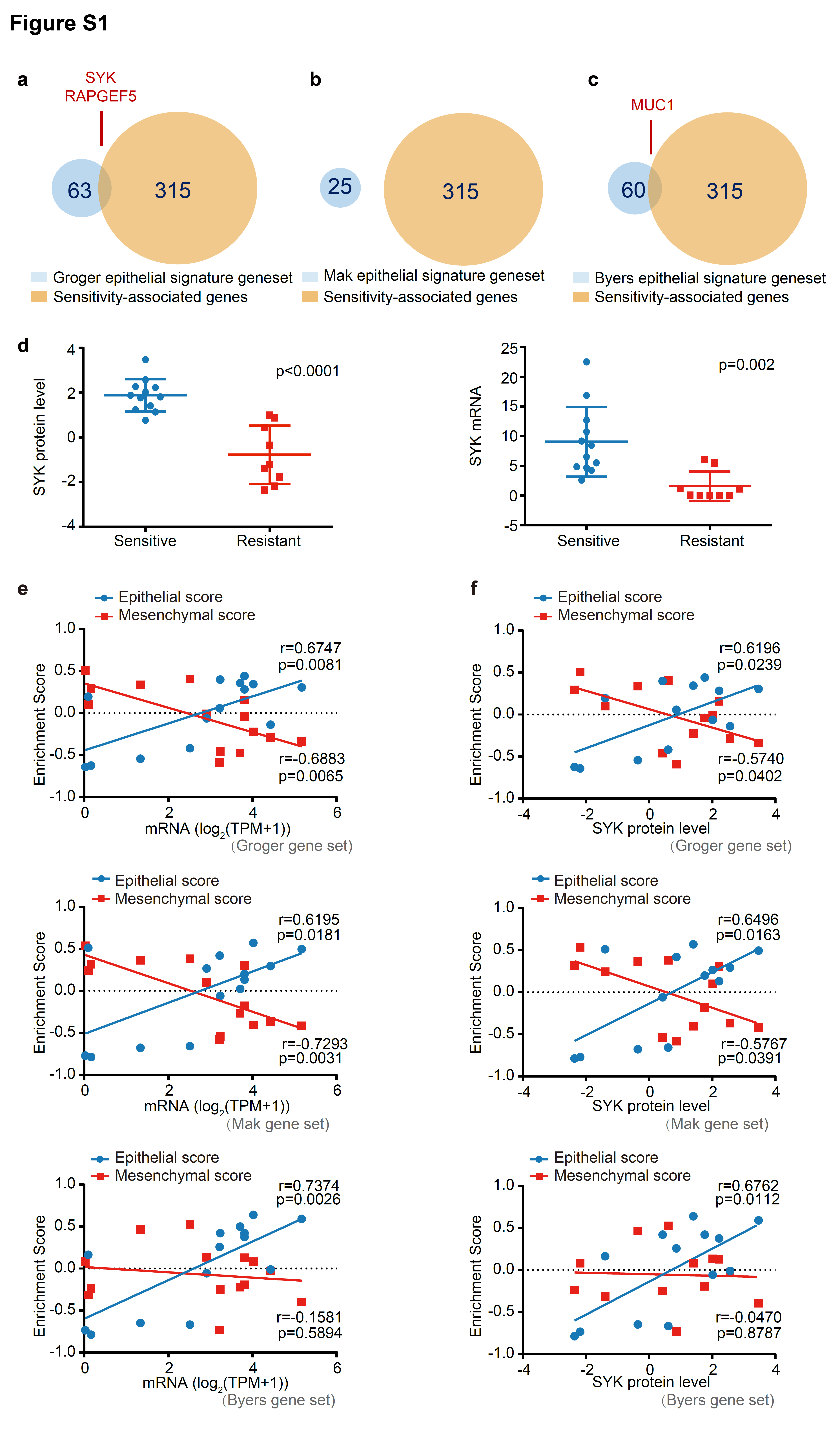


**Figure S1. The sensitivity to kinase inhibitors is related to the SYK expression level and positively associated with the epithelial cell state. a-c** Venn diagram illustrating the overlap of epithelial signature genes with the significant sensitivity-associated genes indicated in Fig. 1**a**. The indicated epithelial signature gene sets are shown in the individual panel. **d** Comparison of SYK protein (left panel) and mRNA (right panel) levels in the sensitive group versus the resistant group indicated in Fig. 1**a** (determined according to the IC_50_). **e** and **f** Pearson correlation analysis between E/M enrichment scores and SYK mRNA levels (**e**) or protein levels (**f**) in the cell lines indicated in Fig. 1**a**. The indicated E/M signature gene sets are shown in the individual panel. SYK protein level data for H3122 cells are not available in the CCLE.


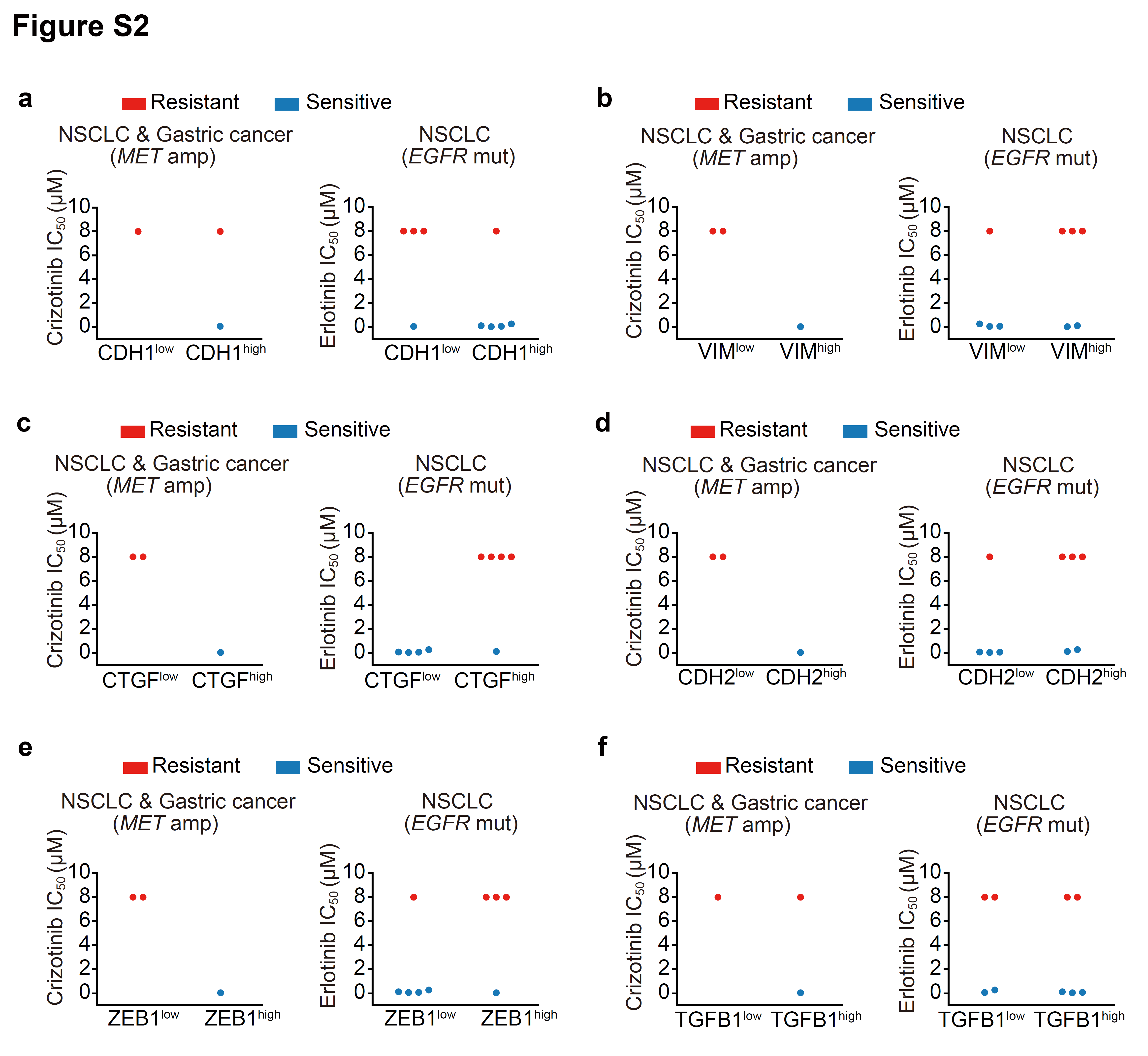


**Figure S2. Some classical E/M signature markers failed to predict a response to c-Metis and EGFRis. a**-**f,** Crizotinib (left) or erlotinib (right) sensitivity was assessed according to the expression of *CDH1* (**a**) *VIM* (**b**) *CTGF* (**c**) *CDH2* (**d**) *ZEB1* (**e**) and *TGF-Β1* (**f**) based on data from the CCLE.


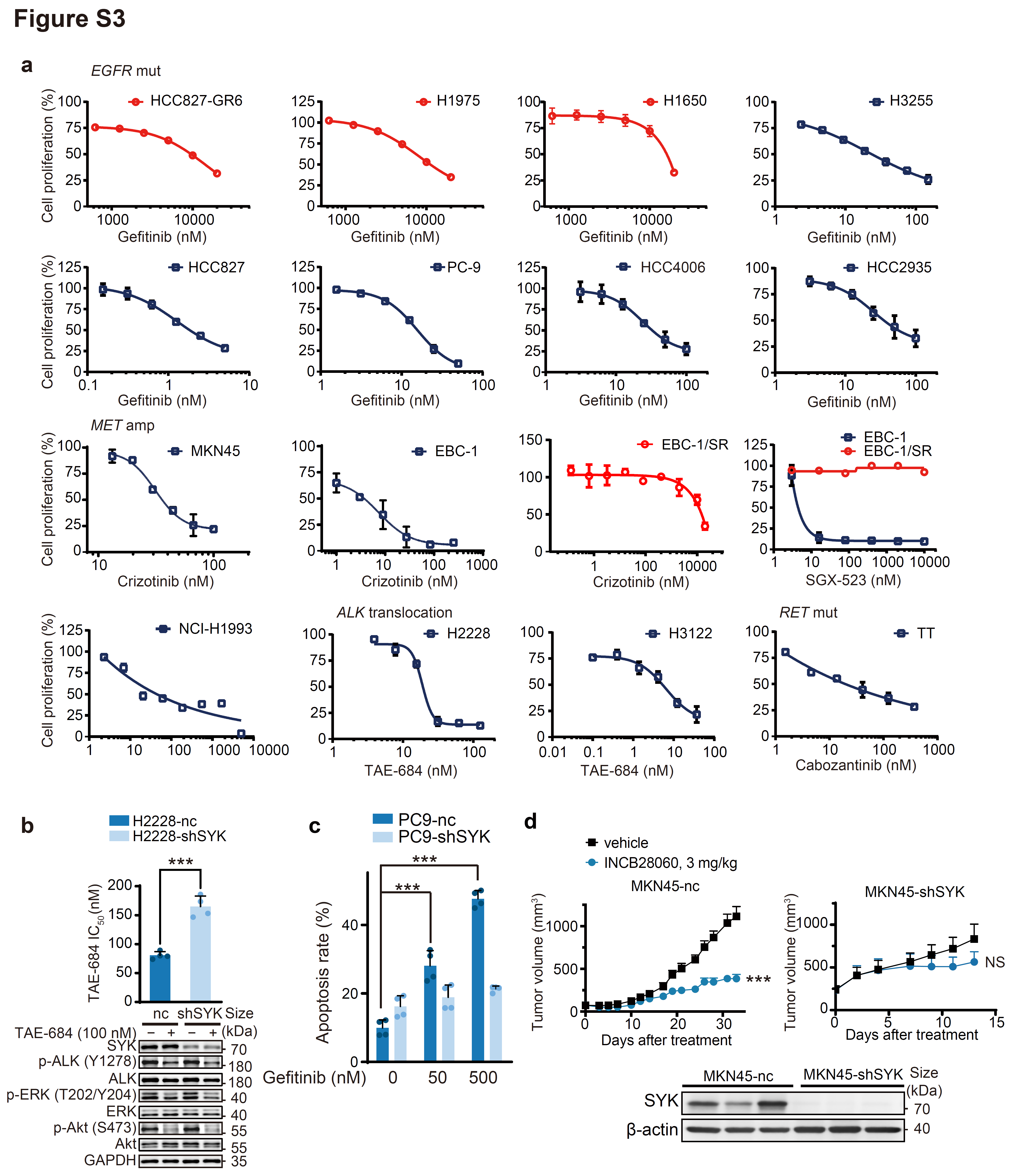


**Figure S3. Drug-response curve of cells treated with the indicated kinase inhibitors and other tests *in vitro* and *in vivo* with cells upon stable SYK knockdown. a** Cells were treated with the indicated kinase inhibitor at a range of concentrations for 72 h, and the IC_50_ values were measured using an SRB assay or the CCK-8 assay (for the TT cell line). Error bars represent the mean ± SD from two to four independent experiments. **b** The change in cell sensitivity upon stable SYK knockdown. Knockdown efficiency and the ALK signaling pathways are shown. **c** The effect of stable SYK knockdown on gefitinib-induced cell apoptosis. Apoptotic cells were detected by annexin V/PI dual staining. **d** The change in the response to INCB28060 in MKN45 xenograft-bearing mice upon stable SYK knockdown (n = 12/group in MKN45-nc model, n = 8/group in MKN45-shSYK model). Data shown are representative results from two to four independent experiments. The data in **a**, **b** and **c** are presented as the mean ± SD; the data in **d** are presented as the mean ± SEM. ***p<0.001, **p < 0.01, and *p < 0.05 using Student’s*t* test in **b** and **d** and using one-way ANOVA in **c**.


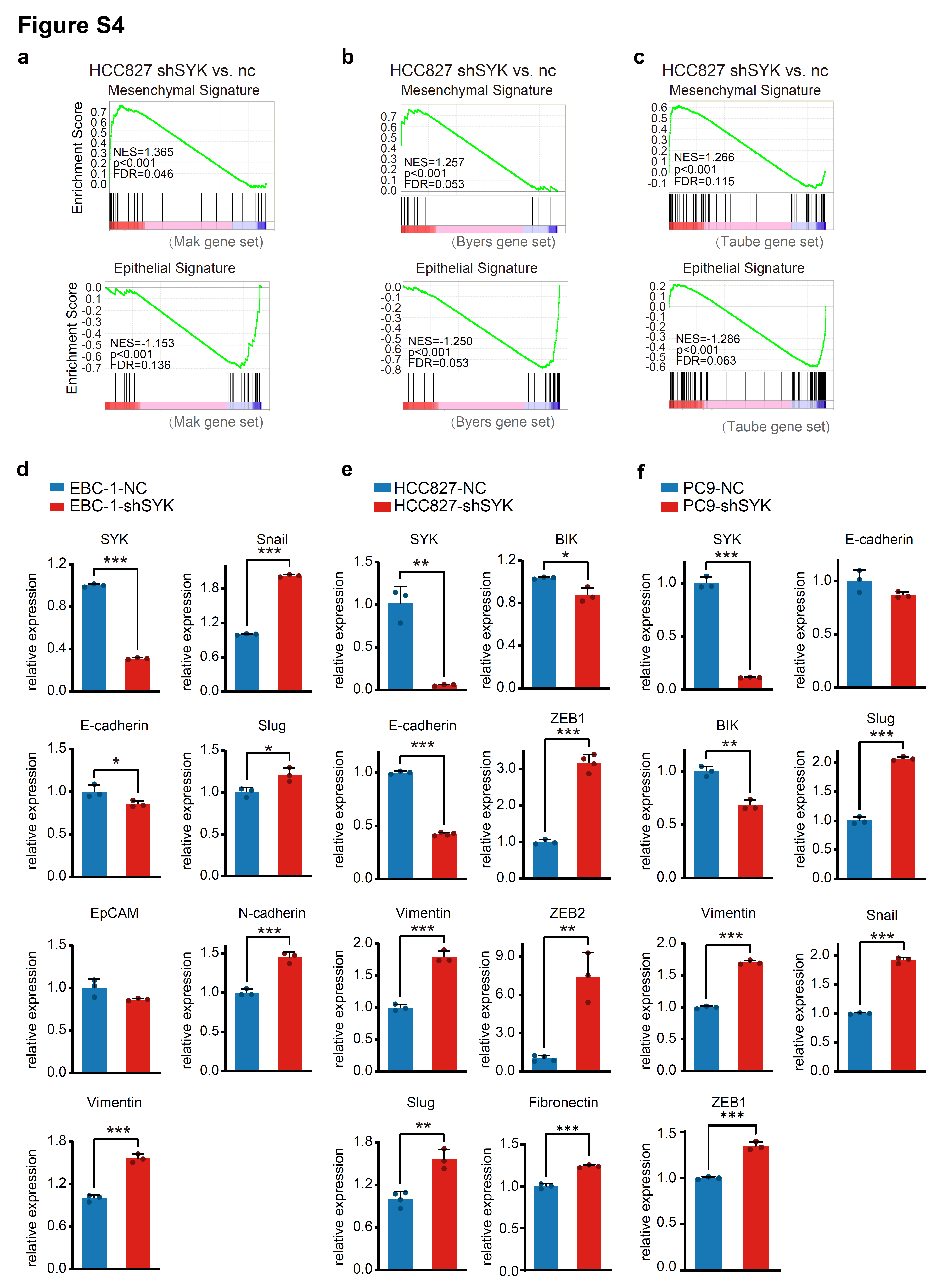


**Figure S4. SYK-depleted cancer cells showed transition from an epithelial state to a mesenchymal state. a-c** GSEA of stable SYK knockdown HCC827 cells versus negative control HCC827 cells. The gene sets shown are indicated as epithelial or mesenchymal state-associated genes. **d**-**f** qRT‒PCR analysis of the representative E/M signature in EBC-1 (**d**) HCC827 (**e**) and PC9 (**f**) cells upon stable SYK knockdown. NS, *p<0.05, **p<0.01 and ***p<0.001, Student’s *t* test. The data shown are representative results from three independent experiments.

**
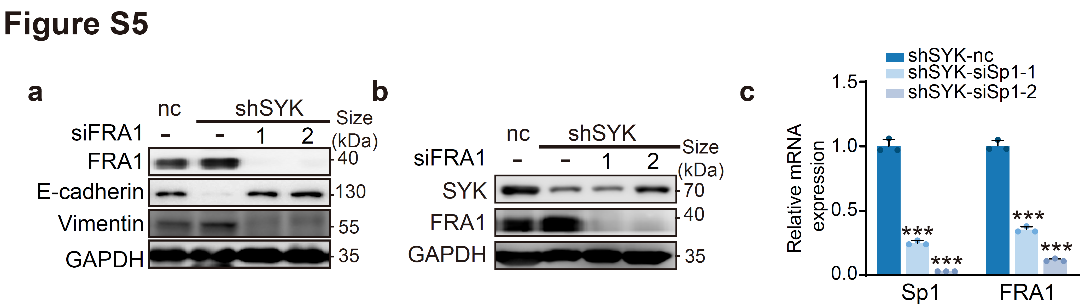
Figure S5. Immunoblot analysis of FRA1 transient knockdown. a** Western blot of E/M markers in PC9-shSYK cells upon FRA1 siRNA transfection. **b** Western blot of FRA1 in MET-amplified EBC-1 cell line with stable SYK knockdown upon further siFRA1 transient transfection. **c** qRT‒PCR analysis of FRA1 in HCC827-shSYK cells upon Sp1 transient transfection. The data in c are presented as the mean ± SD ***p<0.001 using ANOVA. The data shown are representative results from two or three independent experiments.

**
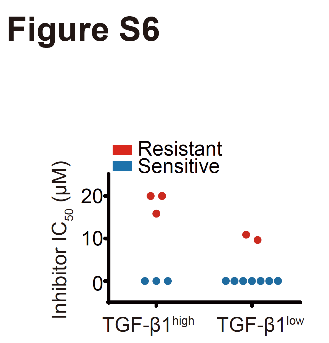
Figure S6. Cell sensitivity to kinase inhibitors was predicted according to TGF-β1 expression in a panel of available cancer cell lines (also shown in Fig. 1f).** The TGF-β1 level was measured, and the cutoff value was determined as described in Fig. 3**d**.


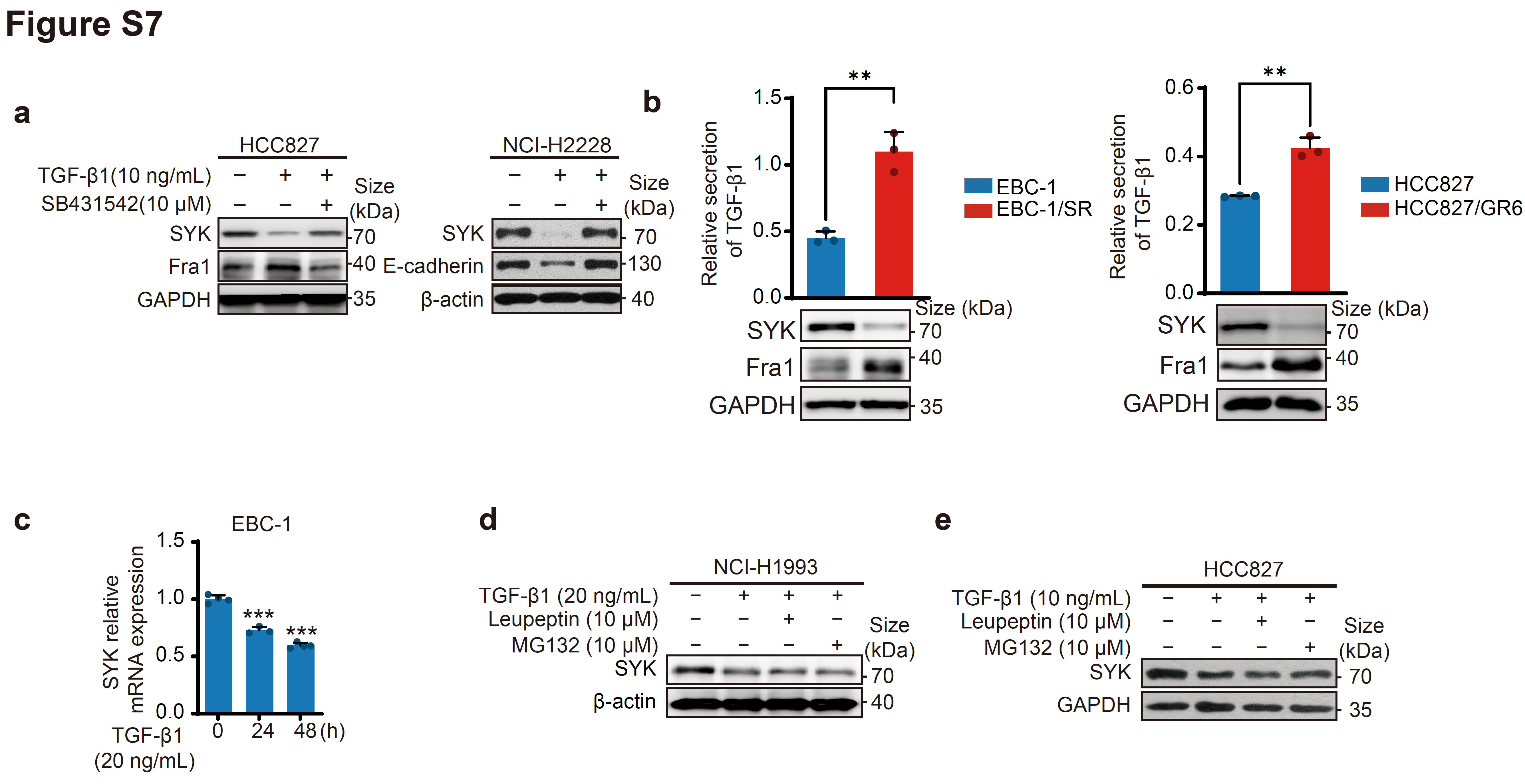
**Figure S7. TGF-β1 negatively regulates SYK. a** Immunoblot analysis of the indicated cell lines treated with TGF-β1 alone or together with a TGF-β1 inhibitor for 96 h. **b** The relative secreted TGF-β1 levels and SYK and FRA1 protein levels in the indicated cell lines. **c** SYK mRNA level alterations in EBC-1 cells treated with TGF-β1. **d** and **e** NCI-H1993 (**d**) or HCC827 (**e**) cells were treated with TGF-β1 (10 ng/mL) alone or in combination with protease inhibitor leupeptin (10 μM) or the proteasome inhibitor MG-132 (10 μM). Leupeptin or MG-132 was added 6 h before the cell lysates were collected, and the cell lysates were then subjected to immunoblotting. ***p < 0.001, **p < 0.01, using Student’s *t* test in **b** and one-way ANOVA in **c**. The data shown are representative results from two or three independent experiments.


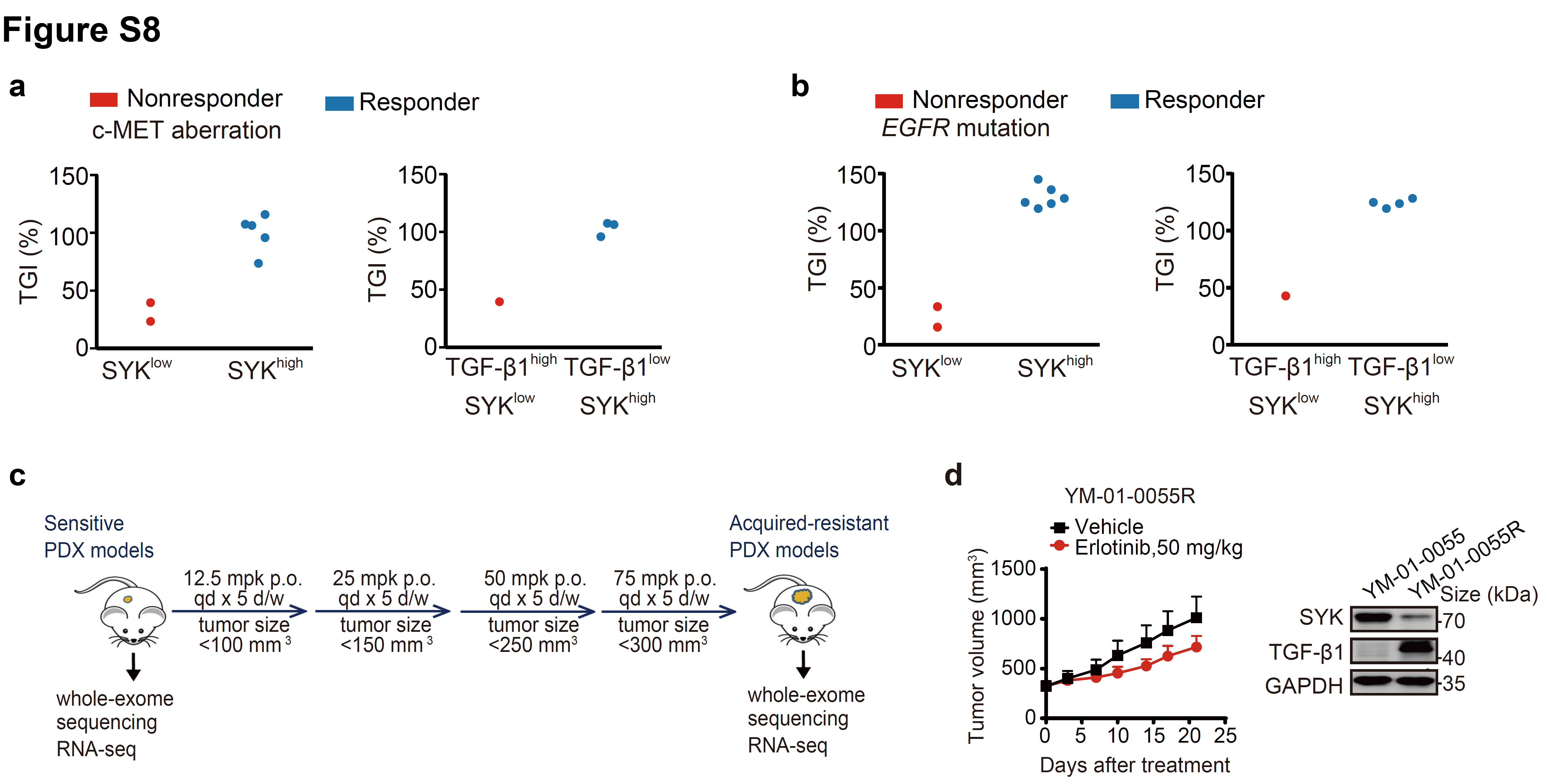
**Figure S8. Test and analysis in PDX models with c-Met aberrations and EGFR mutations. a** and **b** The response to the indicated kinase inhibitors was evaluated according to the TGI rate in PDX models with c-Met aberrations (**a**) and *EGFR* mutations (**b**) stratified according to SYK expression (left) or the SYK/TGF-β1-negative expression pattern (right). **c** Treatment scheme in the PDX models. **d** Tumor volume curves of the acquired resistance models are shown in the left panel. Immunoblotting analysis of intratumoral TGF-β1 and SYK expression in EGFRi-responsive YM-01-0055 cells and the respective PDX models with acquired resistance (right panel).

­­­
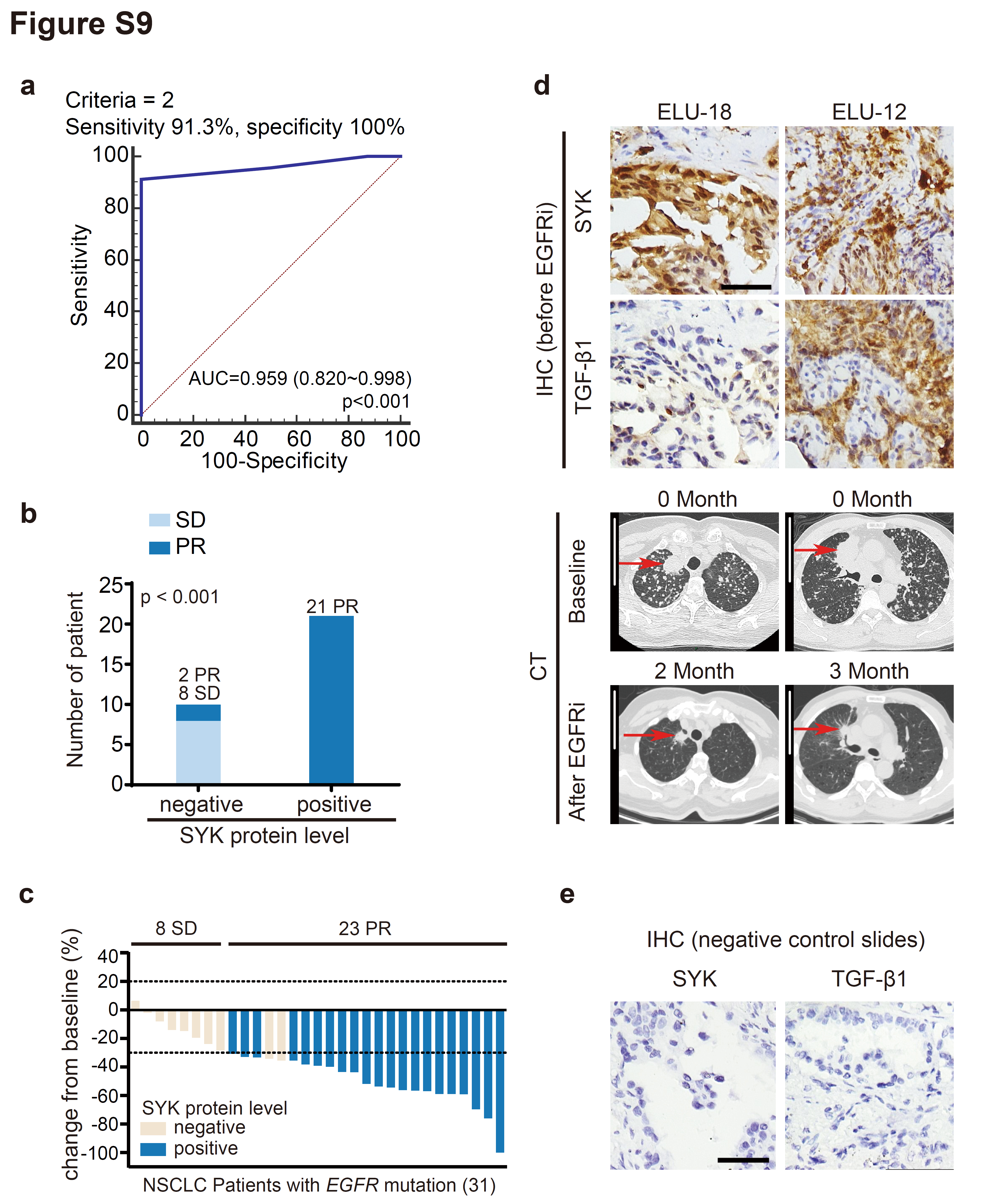


**Figure S9. SYK-positive/EGFR-mutant NSCLC patients uniformly respond to EGFRis. a** ROC curve of SYK levels for identifying an expanded group of EGFRi responders among NSCLC patients with EGFR mutations (n=31). AUC values, shown in parentheses, represent the areas under the respective ROC curves and provide an overall measure of predictive power. **b** The distribution of the therapeutic response in SYK-low and SYK-high patients with EGFR mutations. **c** Waterfall plot of the maximum change from baseline of the longest tumor diameter for patients with evaluable tumors in the EGFR-mutant cohort. **d** Representative SYK and TGF-β1 staining (upper) and chest CT images before and after targeted therapy (lower) in representative patients. Detailed case information is presented in the Methods section. The arrow indicates the location of the tumor. **e** Representative image of negative control slides. Scale bars for the IHC image, 50 μm. Scale bars for the CT image, 10 cm.


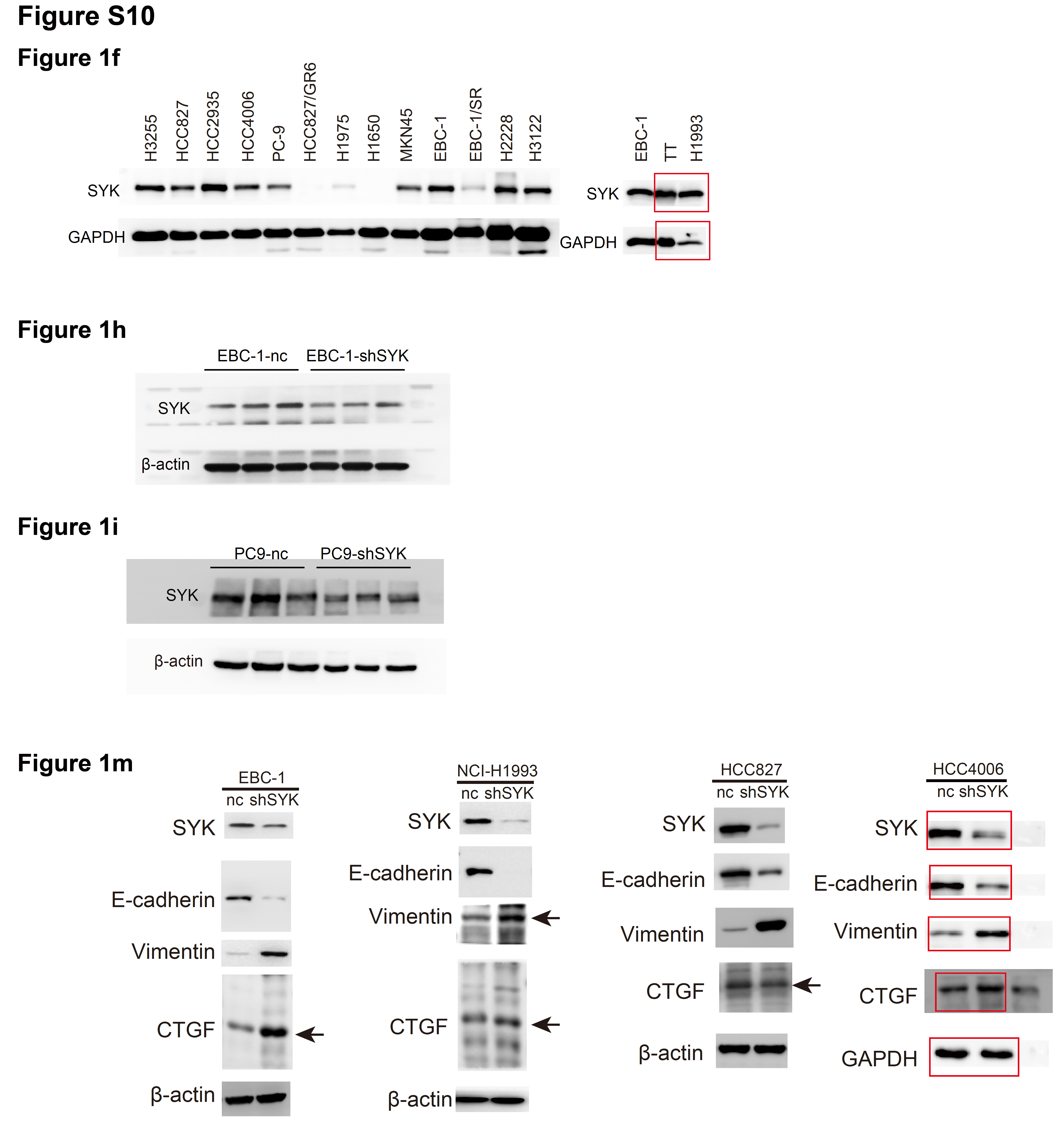


**Figure S10. Raw data of western blot related to Fig.1f 1h 1i and 1m.**


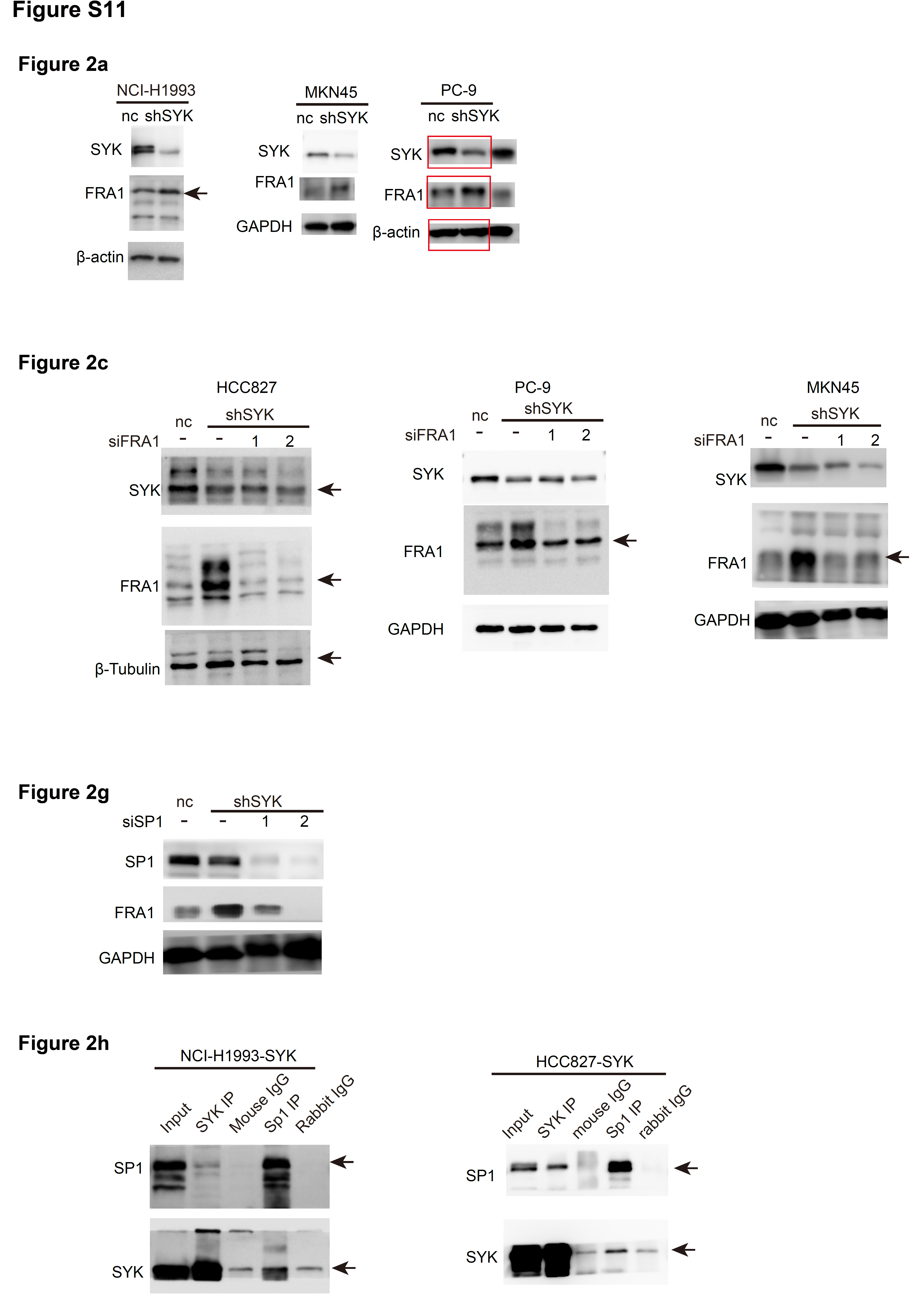


**Figure S11. Raw data of western blot related to Fig.2a 2c 2g and 2h.**


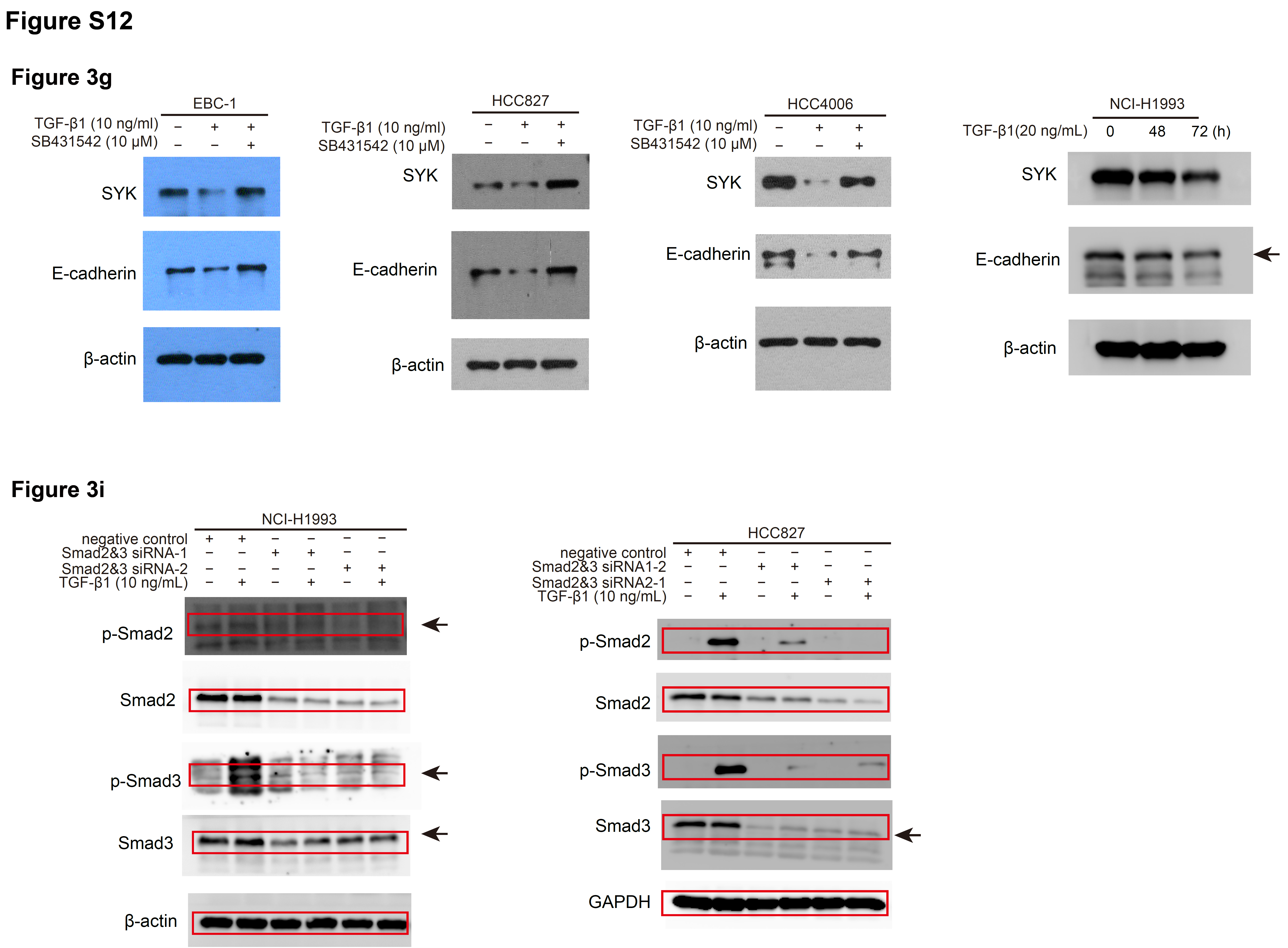


**Figure S12. Raw data of western blot related to Fig.3g and 3i.**


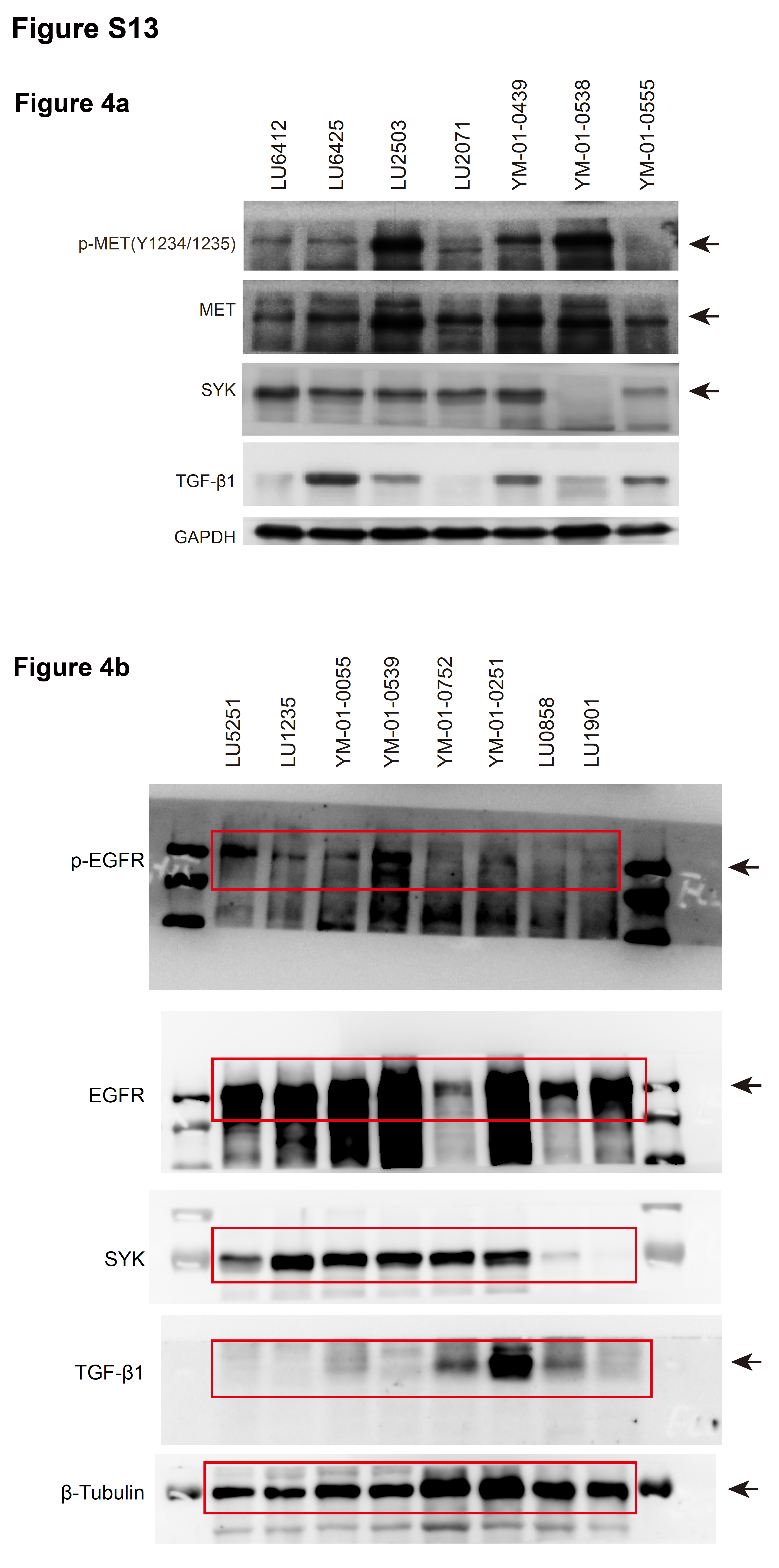


**Figure S13. Raw data of western blot related to Fig.4a and 4b.**

**
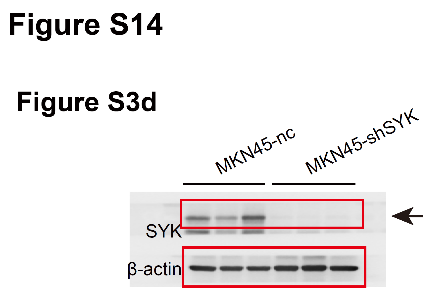
Figure S14. Raw data of western blot related to Fig.S3d.**

**
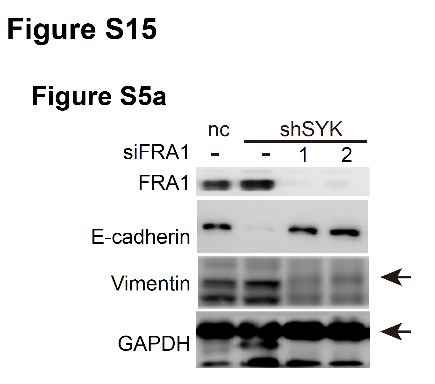
Figure S15. Raw data of western blot related to Fig. S5a.**


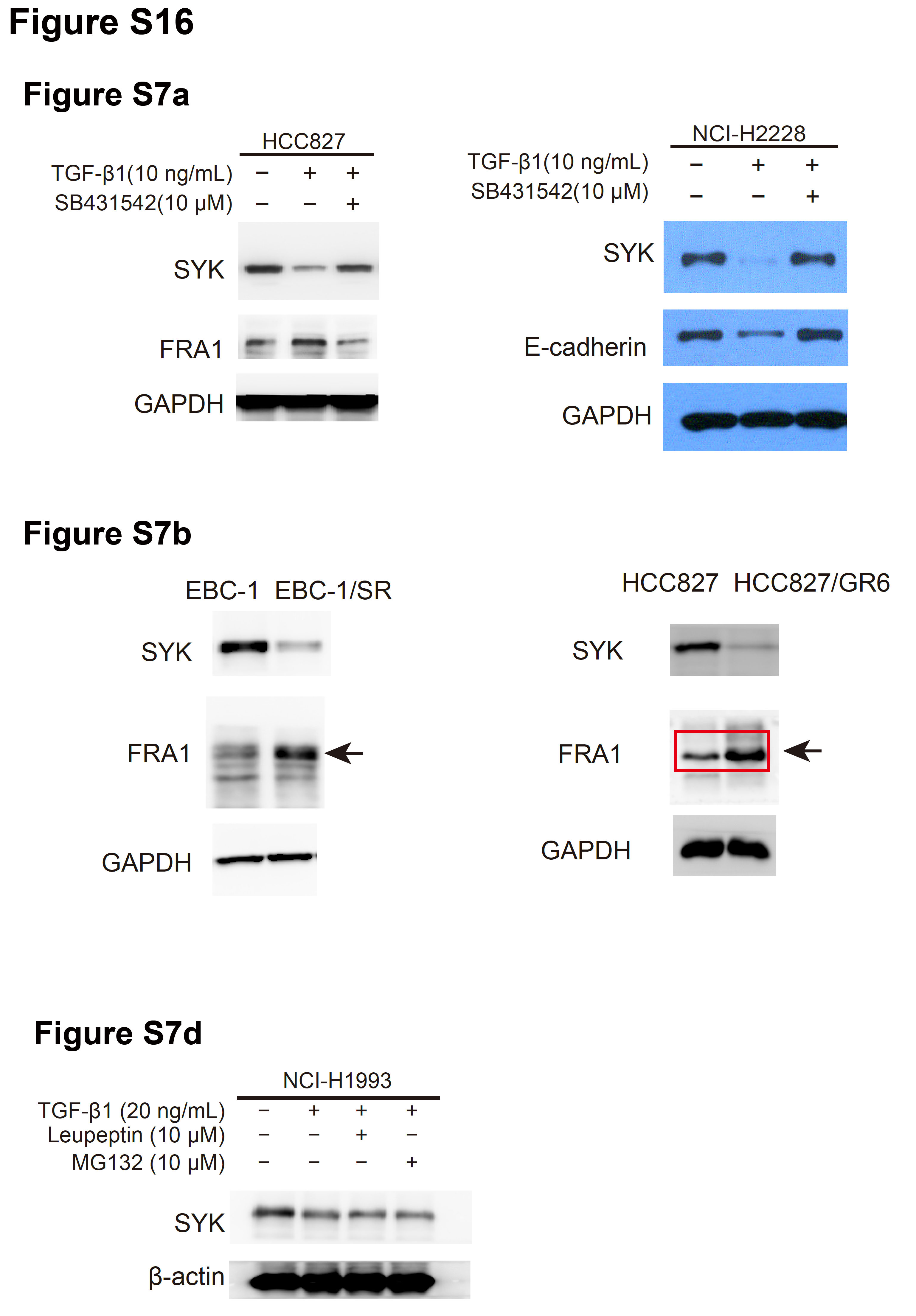


**Figure S16. Raw data of western blot related to Fig. S7a S7b and S7d.**
